# Differential diagnosis of orthostatic dizziness with persistent postural-perceptual dizziness and its underlying mechanisms

**DOI:** 10.3389/fneur.2025.1642869

**Published:** 2025-10-01

**Authors:** Zhang Dao Pei, Zhang Yong Hui, Liu Bing Yang, Zhang Huai Liang, Zhao Min

**Affiliations:** The First Affiliated Hospital of Henan University of Chinese Medicine, Zhengzhou, China

**Keywords:** persistent postural-perceptual dizziness, orthostatic dizziness, differential diagnosis, mechanisms, comorbidities

## Abstract

Persistent postural-perceptual dizziness (PPPD) is characterized by one or more symptoms of dizziness, unsteadiness, or non-spinning vertigo, which persist on most days for at least 3 months. The most common symptom of PPPD worsens when standing or walking, often leading to confusion with other forms of orthostatic dizziness (OD). There are some main differential diagnosis as follows: hemodynamic OD, postural orthostatic tachycardia syndrome, vestibular syncope, BPPV, bilateral vestibulopathy, primary orthostatic tremor, sensory neuropathy, neurodegenerative disorders, cerebral small vessel disease associated with gait disorders, dizziness due to cardiac problems, orthostatic cerebral hypoperfusion syndrome, intracranial hypotension, and the possible mechanisms by which these diseases are associated with OD are briefly elaborated. However, the mechanism underlying OD in PPPD patients remains unclear. There are some impact factors of OD with PPPD, including sex and age, anxiety state and neurotic personality, comorbid vestibular disorders. There are some underlying mechanisms of OD with PPPD, such as altered activity and connectivity of cerebral cortical networks, vestibular-autonomic dysfunction and sensory-perceptual dysfunction, hemodynamic changes, changes in postural control, otolith dysfunction, visual and somatosensory dependence, neurotransmitter abnormalities. For patients with established PPPD, it is important to distinguish the etiologies of OD from other relevant diseases, enabling early intervention and preventing adverse effects on workability, and evaluate responses to therapies to reduce diagnostic errors and missed diagnoses.

## 1 Introduction

Persistent postural-perceptual dizziness (PPPD) is a significant public health concern, accounting for the principal diagnosis in 20% of all dizzy patients in general neurology clinics (1). It is typically associated with moderate-to-severe disability and a poor quality of life (2). PPPD is characterized by one or more symptoms of dizziness, unsteadiness, or non-spinning vertigo, which persist on most days for at least 3 months (3). The most common symptom of PPPD worsens when standing or walking (4), often leading to confusion with other forms of orthostatic dizziness (OD) (5).

OD is a common complaint that causes gait and balance issues, often described as lightheadedness or a feeling of impending fainting with postural changes. It is frequently reported in general practice and is defined as dizziness triggered by a change in body posture, such as moving from lying to sitting or sitting to standing (6). OD accounts for 42% of all dizziness cases and 55% of non-vestibular dizziness diagnoses, with a 12-month prevalence of 10.9% and a lifetime prevalence of 12.5% (6). Statistics show that OD leads to syncope in 19% of affected individuals, falls in 17%, and traumatic injury in 5%, especially occurring in change of severely hemodynamic abnormality (7). In working individuals, OD is associated with a loss of work days in 12% (8). Historically, OD has been attributed to orthostatic hypotension (OH). However, the relationship between spontaneously occurring OD, measured OH, and symptoms of OD during postural testing is complex, as they do not correlate well and do not share the same epidemiological characteristics (9). With an increasing understanding of the various diagnoses related to OD (Table 1), there is a pressing need to improve the identification of underlying etiologies.

**Table 1:** Considerations for the differential diagnosis of orthostatic dizziness with PPPD.

The 12 diagnoses of orthostatic dizziness with PPPD
Hemodynamic orthostatic dizziness (orthostatic hypotension, postural orthostatic tachycardia syndrome)
Vestibular syncope
Benign paroxysmal positional vertigo
Anxiety/depressive disorder
Bilateral vestibulopathy
Primary orthostatic tremor
Peripheral neuropathy
Gait disorder with cerebral small vessel disease
Neurodegenerative disorders
Dizziness due to cardiac problems
Orthostatic cerebral hypoperfusion syndrome
Intracranial hypotension

OD in the context of PPPD is generally considered benign, with somatosensory inputs such as touching fixed objects (e.g., furniture or walls), using gait aids, or holding onto others alleviating symptoms (10). However, many patients with OD suspected PPPD report more severe symptoms when standing or walking compared to sitting or lying down, which may lead to reduced work ability and increase the risk of adverse events, including syncope, trauma, and falls, particularly among elderly patients with comorbid conditions, which is a flag sign diagnosed from PPPD (11). Furthermore, OD can trigger or present as one of the most prominent symptoms, either associated with organic disease or in conjunction with PPPD. On account of this, when diagnosed PPPD with OD, clinicians firstly should be distinguished from hemodynamic orthostatic dizziness, chronic anxiety and depressive disorders, bilateral vestibulopathy, peripheral neuropathy, other clinical or subclinical gait disorders, cardiac problems, orthostatic cerebral hypoperfusion syndrome. Secondly, PPPD with OD also should be distinguished from positional dizziness, which is triggered by a change in the head position with respect to gravity. Therefore, it is essential to perform thorough differential diagnoses of PPPD with OD and better understand the possible mechanisms involved to guide clinical practice. This review aims to summarize the differential diagnosis of OD with PPPD and explore the potential mechanisms of common diseases associated with OD to enhance understanding and management strategies.

## 2 Differential diagnosis of OD with PPPD

### 2.1 Hemodynamic orthostatic dizziness

Patients with hemodynamic OD may experience sensations of veering from side to side when walking, and in severe cases, one may be unable to stand or experience a pre-syncopal state. Detailed history-taking focusing on the patient’s symptoms can help to identify hemodynamic orthostatic dizziness. Clinicians need to ask patients if they are experiencing disequilibrium in their legs or lightheadedness during orthostasis.

#### 2.1.1 Orthostatic hypotension

OH can arise from sympathetic adrenergic failure, which leads to inadequate peripheral vasomotor responses due to insufficient norepinephrine release from sympathetic nerves (neurogenic OH) (12). It is often associated with diabetic and non-diabetic autonomic neuropathy, neurodegenerative diseases such as Parkinson’s disease (PD) or multiple system atrophy (MSA), and primary autonomic failure (13). Additionally, non-neurogenic causes can include medications, hypovolemia, deconditioning, or systemic infections (non-neurogenic OH).

There are various patterns of OH in patients with OD. Blood pressure and heart rate responses vary in representative subjects with classic OH, delayed OH, early OH, and transient OH. Understanding these patterns can assist clinicians in comprehending the autonomic dysfunction mechanisms associated with OD.

#### 2.1.2 Postural orthostatic tachycardia syndrome

Postural orthostatic tachycardia syndrome (POTS) is a common cause of OD. It is characterized by the emergence of orthostatic symptoms alongside a heart rate increase of 30 or more beats per minute upon standing (12). Females are more frequently affected than males. POTS may coexist with PPPD or exhibit similar features. The onset of POTS typically occurs between the ages of 15 and 50 (14).

The pathophysiology of POTS is complex and diverse. Potential causes include a partially denervated circulatory system, a hyperadrenergic state, hypovolemia, peripheral blood pooling, or prolonged bed rest (15). Some patients with POTS possess anti-ganglionic (α3) acetylcholine receptor antibodies, indicating a limited form of autoimmune autonomic neuropathy (16). Additionally, hyperventilation and psychological factors may play a role in POTS development (17). Patients often display overlapping characteristics from several mechanisms.

In hyperadrenergic POTS, the pathophysiology merges with increased sympathetic nervous system (SNS) activity. Common comorbidities, such as deconditioning, autoimmune disorders, and autoantibodies, contribute to the POTS phenotype by entering the pathophysiological cascade at different points (18). Abnormal cerebral blood flow (CBF) is central to POTS pathophysiology, with findings indicating reduced cerebral perfusion, impaired autoregulation, and oscillatory CBF associated with cognitive dysfunction. Altered EEG amplitude modulation may reflect abnormal brainstem physiology (19).

POTS presents ongoing diagnostic and therapeutic challenges for clinicians in various specialties, including cardiology, neurology, and autonomic disorders (20). Enhancing clinician awareness is essential to addressing these challenges. Traditionally, POTS was seen as a peripheral nervous system dysfunction. However, recent studies suggest it should also be regarded as a central nervous system (CNS) disorder (21).

### 2.2 Vestibular syncope

Vestibular syncope is a newly recognized syndrome in individuals diagnosed with OD. Vestibular syncope, often resulted from vertigo-related diseases and also probably accompanied PPPD, and comorbidity is prone to misdiagnosis. This condition involves vertigo-induced hemodynamic changes that lead to syncope following vertigo attacks while in an upright position (22). Vestibular syncope is associated with various vestibular disorders and requires careful evaluation and intervention to prevent recurrent falls and significant injuries.

A study (22) retrospectively analyzed 53 patients with vestibular syncope. Of these, 20 patients (37.7%) experienced multiple episodes of syncope, and seven patients (13.2%) sustained potentially life-threatening injuries. The most common underlying vestibular disorders were Meniere’s disease and benign paroxysmal positional vertigo (BPPV). Abnormal vestibular function tests included impaired cervical vestibular-evoked myogenic potentials and positive head impulse tests. However, many patients exhibited abnormal vestibular function without sufficient evidence to pinpoint specific vestibular disorders.

The dual reflex pathways—vestibulo-sympathetic and baroreflex—suggest that vestibular syncope is a neurally mediated reflex syncope. This is associated with sudden hemodynamic changes during vertigo (23). To ensure proper vestibulo-sympathetic reflex activity, accurately estimating gravitational and inertial accelerations is essential. This estimation relies on the functional integrity of both the velocity-storage circuit and the peripheral vestibular system (24, 25). Future studies with larger sample sizes and more detailed designs will be necessary to further investigate vestibular syncope.

### 2.3 Benign paroxysmal positional vertigo

BPPV should be distinguished from PPPD with OD. In cases of vertical canal BPPV, symptoms can occur not only when sitting up from a supine position but also when lying down from sitting. Conversely, PPPD patients with OD typically present symptoms only upon standing, without issues during other positional changes (26). Positional tests for BPPV should still be conducted in patients with OD, even if their dizziness is not positional (27). A study found that a considerable number of patients with OD were diagnosed with BPPV, accounting for 36.3% of cases (28). One explanation for OD in BPPV may be otolith organ dysfunction. Alternatively, orthostatic intolerance could contribute to OD in these patients. Therefore, it is advisable to conduct orthostatic testing, such as orthostatic vital sign measurements or head-up tilt table tests, as part of the initial evaluation for individuals with simple OD.

On the contrary, OH as a common OD, can influence the recurrence of BPPV. A report indicated that OH may partially impact BPPV recurrence. Among 239 patients studied, 9% experienced a single recurrence, while 3% had multiple recurrences. The prevalence of OH in the overall BPPV patient population was found to be 8.3% (27). Two mechanisms can be considered regarding how OH leads to BPPV. First, a reduction in perilymph due to cerebrospinal fluid (CSF) hypovolemia may cause compensatory endolymphatic hydrops, resulting in vertigo and auditory dysfunction. Second, irritation of the vestibular and cochlear nerves in the internal acoustic canal, caused by venous engorgement, may affect the otolith organ (29).

Another study involving 58 BPPV patients revealed that 25 (43%) experienced residual dizziness after successful canal repositioning procedures. Orthostatic disorders were common among these complaints. The incidence of OH was significantly higher in patients with residual dizziness at follow-up—40% compared to 3% in those without residual dizziness (30). In BPPV, residual dizziness after treatment may be linked to sympathoneural autonomic dysfunction, contributing to OD. This investigation may enhance our understanding of residual dizziness mechanisms in BPPV patients.

These findings suggest that OD could be a complaint among BPPV patients. Additionally, BPPV with OD may arise due to comorbidities with OH, which can occur during acute episodes or as residual dizziness post-treatment.

### 2.4 Chronic anxiety and depressive disorders

PPPD often accompanies chronic anxiety, including generalized anxiety disorder, agoraphobia, social phobia, obsessive-compulsive disorder, depression, and post-traumatic stress disorder (31). Therefore, when patients with chronic anxiety and depressive disorders report lightheadedness upon moving from sitting or supine to an upright position, OD related to PPPD should be considered in the differential diagnosis. Simple self-report questionnaires can effectively identify psychiatric morbidity (12). However, some dizziness patients with autonomic symptoms may not exhibit signs of psychiatric disorders.

A study (32) enrolled patients showing symptoms of autonomic dizziness. The autonomic tests included 45 min of head upright tilt (HUT), followed by 20 min of inhaling 5% CO2, and then another round of HUT, concluding with 2 min of voluntary hyperventilation and one more HUT. Most participants displayed signs of autonomic dysfunction, including abnormal heart rate, blood pressure, or respiratory responses to HUT. However, many of these abnormalities could be overlooked by current autonomic testing methods. Therefore, there may be a need to update autonomic testing protocols to identify clinically relevant issues in patients with dizziness.

Another study focused on patients with PPPD and had an equal number of subjects with other types of dizziness. Participants completed the Dizziness handicap inventory (DHI), hospital anxiety and depression scale (HADS) questionnaires. The DHI indicated significant disability in the majority of patients, whereas HADS revealed considerably higher levels of pathological anxiety in the PPPD group (33). Most patients in both groups experienced mild anxiety; however, those with pathological anxiety were more prevalent in the PPPD group. We can consider only those with pathological anxiety as predisposed to developing PPPD. Nevertheless, a positive screen for anxiety or depression does not exclude an underlying disorder related to PPPD or other symptom causes, as psychiatric disorders frequently co-occur with medical issues. Heightened anxiety about postural stability leads to co-contraction of anti-gravity muscles, resulting in increased sensitivity of sensorimotor balance reflexes and rigid control of body sway. This causes a subjective sense of imbalance, which further intensifies anxious control of posture (34).

### 2.5 Bilateral vestibulopathy

Bilateral vestibulopathy (BVP) is a chronic vestibular syndrome marked by postural imbalance and unsteadiness of gait due to vestibular hypofunction. However, some patients with bilateral vestibular hypofunction may not exhibit clinical symptoms of BVP, which only present with OD, it is very easy to be confused with PPPD (35). Typically, BVP patients experience no symptoms whereas sitting or lying down in static conditions, as they do not heavily rely on the vestibular system in those positions. However, walking or making quick head or body movements can trigger blurred vision or oscillopsia (36). A bilaterally reduced or absent angular vestibulo-ocular reflex (VOR) function is critical for diagnosing BVP. Symptoms such as dizziness or unsteadiness tend to worsen in dark environments or on uneven ground for patients with BVP, unlike those with other disorders, especially hard to distinguish PPPD (36).

When vestibular hypofunction is suspected, it is advisable to begin with the video head impulse test (vHIT). This test has a low burden on the subject (37). If the vHIT results are abnormal, further vestibular testing is unnecessary. However, if the vHIT results are normal, caloric testing may be recommended. This is because caloric testing can be more sensitive than vHIT for detecting vestibular hypofunction in certain disorders, particularly Menière’s disease (38). Additionally, there may be a dissociation between caloric testing and vHIT, especially in cases of endolymphatic hydrops due to altered inner ear mechanics (39). For BVP, rotatory chair testing can be included to enhance testing specificity (though not sensitivity) and to assess residual vestibular function. Responses to rotatory chair testing are often better preserved than those from vHIT or caloric stimulation (37).

The etiology of BVP varies based on clinical course and associated findings. Genetic abnormalities are increasingly recognized in both isolated and complicated forms of BVP (40). Recent advancements in vestibular function evaluation have significantly improved BVP detection, and the introduction of consensus diagnostic criteria by the Barany Society has facilitated related research. BVP can occur in various neurodegenerative disorders affecting the cerebellum or brainstem, such as spinocerebellar ataxia (SCA) and MSA (40). CANVAS (Cerebellar Ataxia, Neuropathy, and Vestibular Areflexia Syndrome) is characterized by late-onset ataxia, sensory neuropathy, and BVP (41). Genetics is playing an expanding role in identifying previously unknown causes of BVP. Vestibular prostheses may improve vestibular function, posture, gait, and quality of life for patients with BVP, thereby broadening therapeutic options in the near future.

### 2.6 Primary orthostatic tremor

Primary orthostatic tremor (OT) is marked by unsteadiness whereas standing, caused by a high-frequency (14–18 Hz) tremor in the legs or arms during weight-bearing (42, 43). In a population referred to a dizziness and balance clinic, primary orthostatic tremor accounted for 0.7% of patients. Thus, it may be a more common cause of dizziness than previously thought. It is also an important differential diagnosis for psychogenic dizziness and PPPD, both of which are characterized by subjective unsteadiness (12, 44).

PPPD with OD may be accompanied by tremor during upright posture, while OT can cause a sensation of imbalance while standing. These features should be considered in the differential diagnosis of OD in PPPD. Notably, OT symptoms improve quickly when patients sit or walk. In some cases, the urge to sit or move may be so intense that individuals with OT avoid situations requiring them to stand still (42). OT can be diagnosed swiftly using Fourier frequency analysis of signals from a posturography platform or superficial electromyography (45).

Functional imaging studies suggest that abnormal activation of the ponto-cerebello-thalamo-motor cortex may underlie primary OT (46). However, the predominant evidence supports the notion of a central oscillatory network involving the cerebellum and its connections (47).

**Figure 1:**
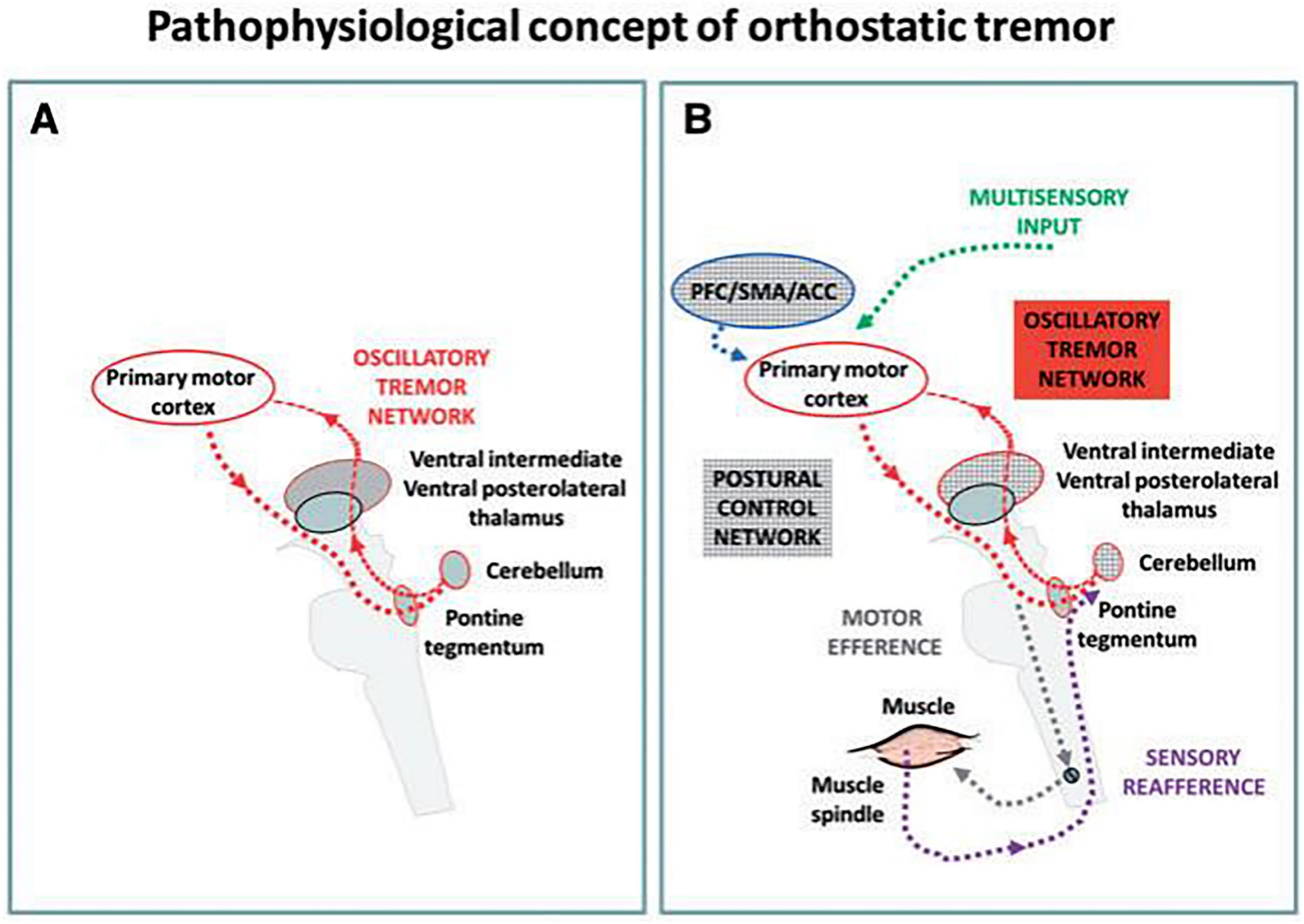
A hypothetical pathophysiological concept of an orthostatic tremor oscillatory circuit and its modulation during upright stance and locomotion (48). **(A)** During the resting condition an intrinsic oscillatory circuit exists but there is no apparent clinical tremor (red). **(B)** Upright stance triggers the network oscillations and consequently causes clinical signs and symptoms. PFC, prefrontal cortex; ACC, anterior cingulate cortex; SMA, supplementary motor area.

Research has identified consistent ponto-cerebello-thalamo-primary motor cortical activations in OT patients (48), observed both at rest and during standing. Variations in neuronal excitability during upright posture might act as triggers for activating the OT circuit. This circuit includes the pontine brainstem tegmentum, the cerebellum, the ventral intermediate and posterolateral thalamic nuclei, and the primary motor cortex bilaterally. All these components may exhibit oscillatory behaviors, but the pontine tegmentum is likely crucial for tremor generation, especially compared to other tremor disorders (Figure 1) (48).

### 2.7 Sensory neuropathy

Patients with peripheral sensory neuropathies often report OD, and it needs to be differentiated from PPPD, especially feelings of unsteadiness, which sometimes appear more severe than the underlying neuropathy (49). Even when the loss of balance seems disproportionate, most individuals with significant large fiber peripheral neuropathy exhibit distal paresthesia, sensory deficits, and diminished ankle reflexes. Nerve conduction studies, Romberg tests, and measurements of blood pressure and heart rate during position changes can help differentiate between sensory neuropathy and OD (50). Additionally, dizziness due to postural imbalance may coexist with PPPD and OH when sympathetic vascular fibers are affected.

A study shows that vestibular dysfunction occurs in 53.2% of individuals with peripheral sensory neuropathy (51). This raises the question of whether the pathological process affecting peripheral sensory nerves may also impact the vestibular nerve. This possibility is supported by the similarity in histologic structure between the vestibular nerve and peripheral sensory nerves. In patients with type 2 diabetes mellitus receiving primary healthcare, utricular function may be impaired even if they do not seek care for sensory or balance decline. This can occur despite the absence of horizontal canal dysfunction or a history of falls (52). Thus, it is crucial to consider abnormal vestibular function in patients with type 2 diabetes mellitus who report dizziness, alongside peripheral neuropathy.

### 2.8 Neurodegenerative disorders (PD and MSA)

Dizziness and disequilibrium are common in PD and MSA. However, early diagnosis of these neurodegenerative diseases are challenging. When patients seek medical attention with chief complaints of unsteady gait or dizziness, they are prone to being misdiagnosed with PPPD. Both Parkinsonian gait and cerebellar ataxia can coexist in patients with OD, as PD and MSA are major causes of neurogenic OH (53). However, neurodegenerative disorders might compromise vestibular function, possibly influencing the symptoms seen in PD and MSA rather than being directly associated with OH.

The integrity of the VOR in patients with PD and MSA needs further investigation (54). During vHIT, reversed and perverted catch-up saccades were more frequent in MSA than PD. Additionally, the gain difference between the anterior and posterior canals was greater in MSA and positively correlated with disease duration (55). Both PD and MSA patients exhibited poorer pure-tone audiometry (PTA) thresholds at high frequencies. In PD patients, cVEMPs were absent bilaterally in 46.7% and unilaterally in 13.3%. For MSA patients, cVEMPs were absent bilaterally in 26.7% and unilaterally in 40% (56). A significant inverse association between disease duration and cVEMP amplitude was found in MSA patients. High-frequency hearing loss and cVEMP abnormalities frequently occur in both MSA and PD, indicating potential audio-vestibular dysfunction even in the absence of reported auditory or vestibular symptoms. These findings suggest that auditory and vestibular dysfunction should be recognized and investigated as nonmotor features in both diseases.

### 2.9 Cerebral small vessel disease associated with gait disorders

Cerebral small vessel disease (CSVD) is a notable contributor to imbalance and falls in the elderly (57). The incidence of dizziness symptoms in patients with CSVD ranges from 35% to 44% (58, 59), which usually manifests as exacerbated symptoms during walking, and is very easy to be misdiagnosed as PPPD.

The frequency of severe lesions with white matter hyperintensities (WMH) at Fazekas stage 3 was notably higher in patients experiencing ‘unexplained’ dizziness compared to those with ‘explained’ causes of dizziness (60). The increased severity of WMH in unexplained cases suggests that these abnormalities may contribute to the onset of dizziness. White matter lesions may cause such dizziness by either inducing a degree of objective unsteadiness or through a disconnection syndrome affecting vestibular or locomotor brain areas.

**Figure 2:**
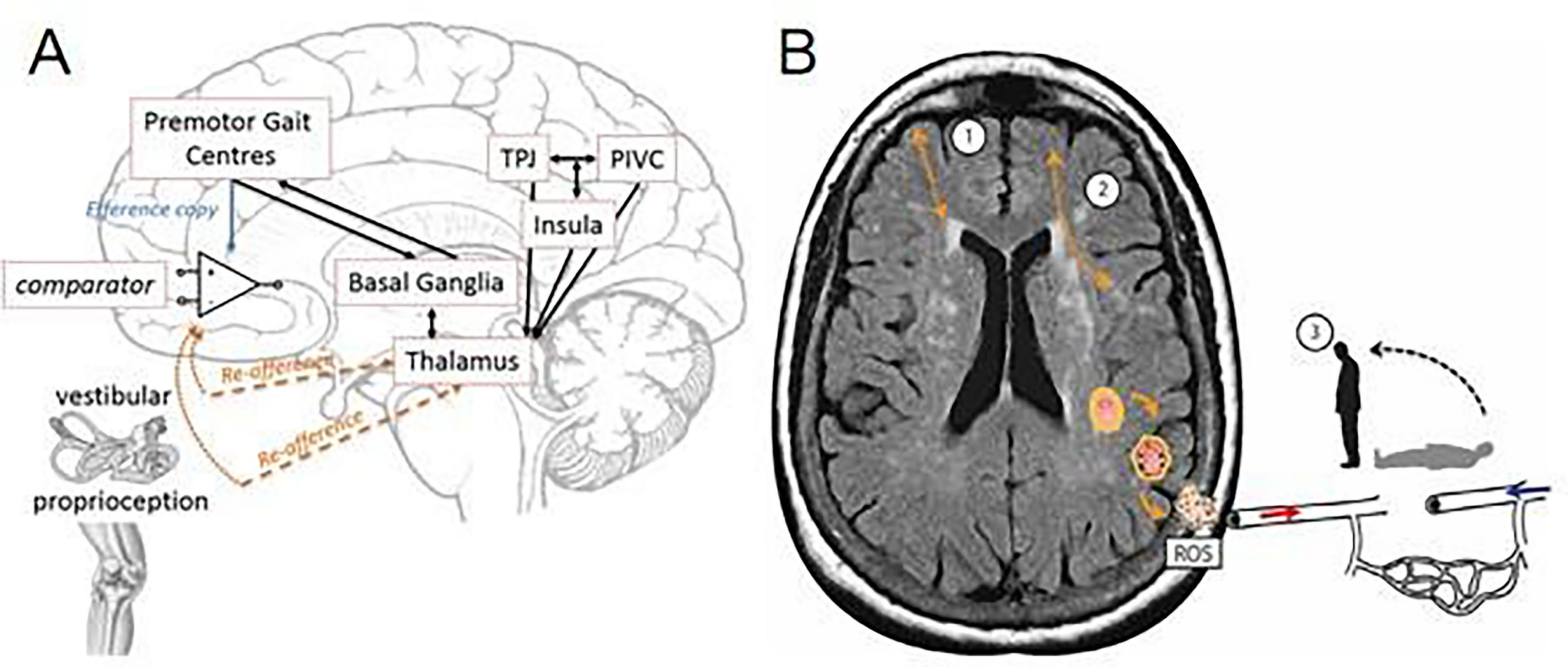
Cerebral hemispheres leading to spatial disorientation and a “vague” feeling of dizziness. **(A)** Theoretical framework underpinning of interconnecting premotor gait centers and basal ganglia networks (cortico-basal-thalamo-cortical loops) (61). **(B)** Schematic of cerebral small vessel disease associated dizziness. In addition to cortico-subcortical (1), and cortico-cortical (2) disconnectivity, localized oxidative stress processes damage the cerebral vasculature, which disrupts cerebral autoregulation leads to intra-cerebral orthostatic hypotension and perfusion, manifest as postural light-headedness and imbalance. TPJ, temporoparietal junction; PIVC, parieto-insular-vestibular cortex; ROS, reactive oxidative species.

Damage to interconnecting premotor gait centers and basal ganglia networks, crucial for balance and movement, results in altered gait and stability. Disrupted connectivity between vestibulo-spatial and vestibulo-motion centers (such as the temporoparietal junction, parieto-insular vestibular cortex, and insula) across the cerebral hemispheres can lead to spatial disorientation and a vague sensation of dizziness (Figure 2) (61). Localized oxidative stress processes can damage cerebral blood vessels, causing endothelial dysfunction and promoting neurodegenerative changes in brain tissue due to reactive oxidative species. This microvascular dysfunction disrupts cerebral autoregulation (62), leading to intra-cerebral OH and perfusion issues, which manifest as dizziness.

### 2.10 Dizziness due to cardiac problems

Dizziness may affect more than half of patients with cardiovascular issues, such as cardiogenic embolism or hemodynamic abnormalities, and can occur in isolation (63), when cardiac symptoms are not obvious or atypical, it can easily be misdiagnosed as PPPD. Approximately 10% of patients with acute myocardial infarction report dizziness as a dominant or presenting symptom (64). Dizziness related to cardiac problems may occur during exertion or when supine, often accompanied by palpitations, chest discomfort, or dyspnea. Patients may have a family history of unexplained sudden death at a young age, structural heart disease, coronary artery disease, or arrhythmias (65), and these symptoms and medical history are helpful for differentiating from PPPD.

Early identification of cardiogenic vertigo (CV) is crucial to prevent serious complications associated with cardiovascular disease. However, the existing literature is limited to case reports, which lack detailed clinical features or diagnostic criteria. In a study of 27 patients with CV, recurrent vertigo occurred without syncope in 52% of cases, whereas it preceded or followed syncope in others (66). The most common cardiac abnormality during vertigo episodes was bradyarrhythmia. Onset age, vertigo duration, accompanying symptoms, and underlying cardiac conditions can help differentiate CV from other vestibular disorders. Early recognition of CV can reduce morbidity and mortality associated with cardiac syncope.

Whether CV occurs in isolation or with syncope may depend on factors such as the duration and extent of brain hypoperfusion, individual variations in cerebral vasculature, and differences in susceptibility to decreased perfusion (67). Presyncopal dizziness typically indicates generalized cerebral ischemia due to a cardiovascular cause. However, the mechanism behind CV remains unclear. A study noted downbeat nystagmus during a presyncopal attack with vertigo in a patient with recurrent asystole due to sick sinus syndrome (68). Given the recurrent isolated vertigo with no other neurological symptoms and the presence of pure downbeat nystagmus, the CV in this patient was more likely caused by cerebellar ischemia, rather than labyrinthine or brainstem ischemia (69). These findings suggest that primary cardiovascular disorders can lead to true vestibular vertigo, often presenting as dizziness.

### 2.11 Orthostatic cerebral hypoperfusion syndrome

OD without OH is common, but its underlying pathophysiology is poorly understood. Orthostatic cerebral hypoperfusion syndrome (OCHOs) is a novel condition characterized by low orthostatic CBF velocity presenting with OD, which is extremely difficult to differentiate this from PPPD. However, clinically, many patients with PPPD accompanied by OD have experienced treatment for cerebral ischemia, and the therapeutic effect is unsatisfactory for most of patients. OCHOs is defined by (1) an abnormal orthostatic decrease in CBF velocity (CBFv) during a tilt test and (2) the absence of OH, arrhythmia, vascular abnormalities, or other causes of abnormal orthostatic CBFv (70).

**Figure 3:**
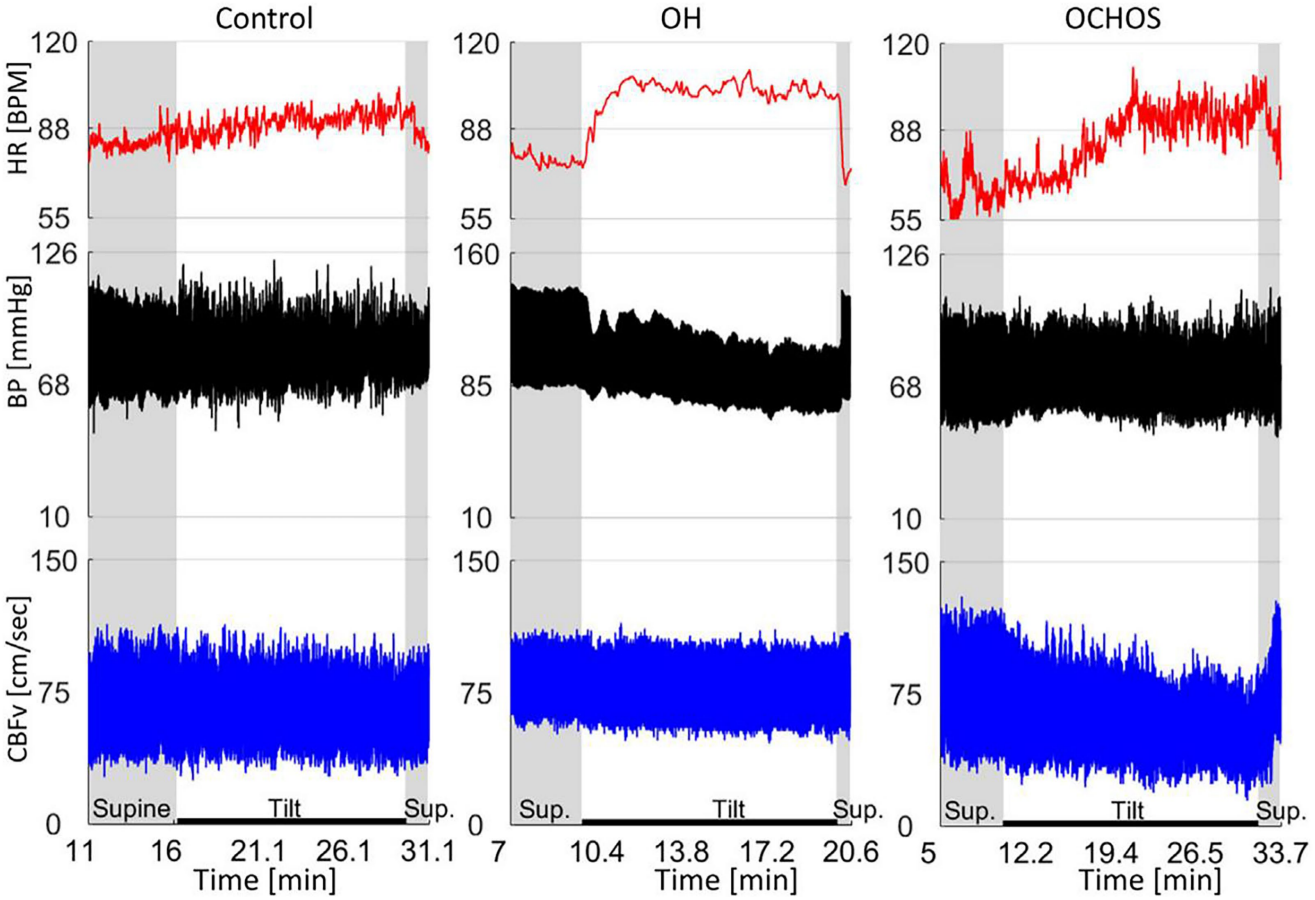
Representative examples of normal orthostatic blood pressure and cerebral blood flow velocity (CBFv) (left panel), orthostatic hypotension (OH) with stable CBFv during tilt test (middle panel), and orthostatic cerebral hypoperfusion syndrome (OCHOs) (right panel) (71).

A retrospective study of 1,279 patients referred for evaluation of unexplained OD found that 102 (7.8%) met the criteria for OCHOs, with mean CBF velocity decreasing without OH (71). Two main pathophysiological mechanisms have been proposed: active cerebral vasoconstriction and passive increase in peripheral venous compliance (72, 73). Detection of OCHOs is straightforward with simultaneous monitoring of hemodynamic variables and CBFv during the tilt test. The concept of OCHOs, which presents as OH with stable CBFv during the tilt test, offers a physiologically plausible mechanism for unexplained OD, which is very common (Figure 3) (71).

Several post-infectious, presumably autoimmune, complications of COVID-19 affecting the brain or peripheral large nerve fibers have been reported. Post-COVID-19 patients may develop chronic fatigue, OD, and brain fog, who be treated according to the scheme of PPPD and the therapeutic effect is discontented, however, some patients are consistent with OCHOs and have responded to immunotherapy with intravenous immunoglobulins (74). Further studies are needed to confirm the association between OCHOs and COVID-19, as well as the benefits of immunotherapy.

### 2.12 Intracranial hypotension

Intracranial hypotension is characterized by orthostatic headache, with some patients presenting OD, which should be differential diagnosis with PPPD, especially in patients with mild symptoms and a long disease course. Intracranial hypotension often induces audiovestibular impairments due to low CSF volume caused by spontaneous or post-traumatic dural lacerations (75, 76).

Intracranial hypotension has been associated with neurotological symptoms, including dizziness (30%), tinnitus (20%), aural fullness (20%), and hearing loss (3%) (77), with another study reporting auditory symptoms in approximately 70% of patients (78). In addition to endolymphatic hydrops and irritation of the vestibulocochlear nerve, compression or traction of the brainstem or cerebellum due to loss of CSF buoyancy may contribute to frequent spontaneous or positional vertical nystagmus in patients with intracranial hypotension (79). BPPV was detected in 11.54% of patients with spontaneous intracranial hypotension, which may contribute to OD (80).

**Figure 4:**
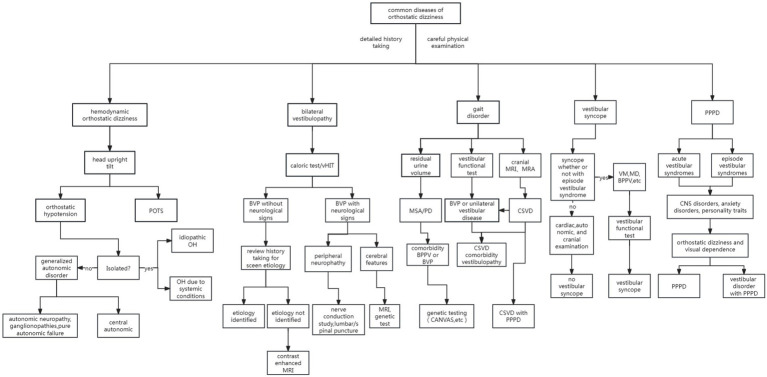
Algorithm to help diagnose the common OD.

The summarizing diagnosis, treatment, prevention, and prognosis of differential diagnosis from PPPD with OD is provided with Supplementary Table 1, along with an algorithm to assist in diagnosing common OD in Figure 4.

## 3 The underlying mechanisms of OD with PPPD

**Figure 5:**
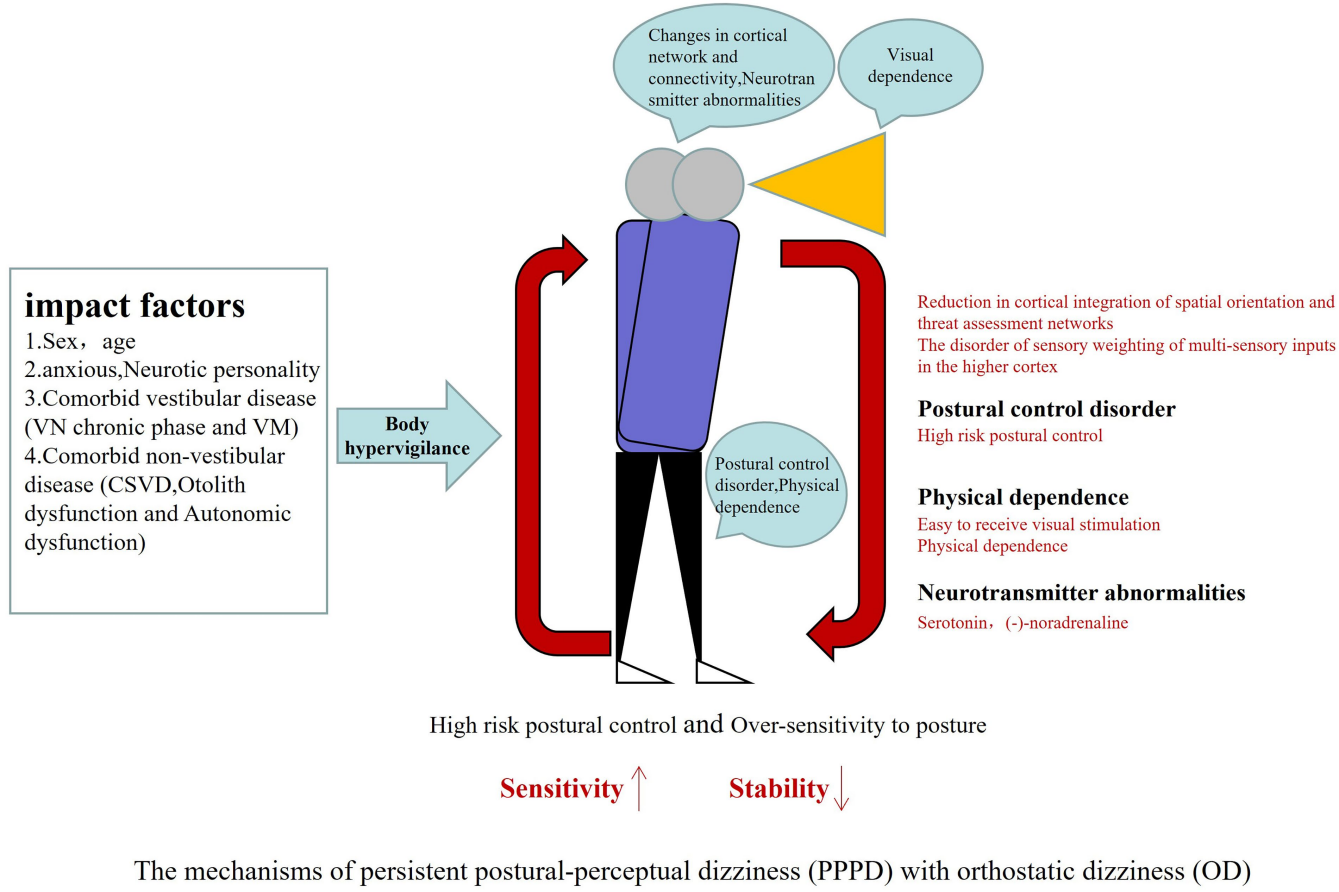
PPPD often have acute vestibular disease as triggering events leading to brain network mismatch, body excessive vigilance, abnormal spatial positioning, visual dependence and high anxiety, coupled with otolith dysfunction and autonomic dysfunction, increasing the strengthening cycle of postural dizziness, when upright or walking demonstrated OD. PPPD, persistent postural-perceptual dizziness; OD, orthostatic dizziness.

OD is common in patients with PPPD, often leading them to avoid going out, walking, or fearing falls. If effective treatment is available, it will be of great significance for improving the quality of life with PPPD patients. However, the mechanism underlying OD in PPPD patients remains unclear. The present review also discussed the underlying impact factors and current understanding of probable pathogenesis of OD with PPPD in this section. The pathogenesis is summarized as shown in Figure 5.

### 3.1 Impact factors of OD with PPPD

The most common aggravating factors for OD in PPPD are physical exertion or exercise (53%) and environmental warming (32%) (1). Physical activities that provoke symptoms are typically routine, such as climbing stairs or doing housework. Environmental warming includes activities on hot days, taking hot showers, or immersion in hot tubs. Postprandial symptom aggravation is recognized in 24% of patients (10), whereas only 6% report symptom aggravation during specific periods of their menstrual cycle (2).

#### 3.1.1 Sex and age

Sex and age influence the occurrence of OD in PPPD. Patients with PPPD tend to be older, with those over 40 significantly outnumbering those under 40 (81). The male-to-female ratio is 1/5.7, with a significantly higher prevalence in females (82). Sex differences in dizziness occurrence can be attributed to the influence of sex hormones, which affect postural dizziness via peripheral sympathetic and parasympathetic activity, as well as central adrenergic and serotonergic systems, vascular tone, and adrenergic control (83). Age-related differences occur because the postural control system degenerates as people age. Maintaining an upright posture requires integration of vision, proprioception, and vestibular input in the central neurological system. As people age, postural control is impaired by various factors (84), including loss of ankle position sense, increased vibration perception thresholds, decreased visual function (e.g., visual acuity, depth perception, contrast sensitivity, and peripheral vision), and reduced vestibular function. Wiesmeier et al. (85) analyzed postural control in 20 healthy elderly individuals and found that compared to younger individuals, the elderly had significantly larger spontaneous sway amplitude and speed, with higher swing frequency. Aging leads to increased reliance on proprioception, decreased feedback loop gain, and increased sensory-motor time delays. Additionally, cognitive decline with age may increase the risk of postural instability. Lamoth et al. (86) found that gait variability in cognitively impaired older adults was significantly greater than in cognitively intact older adults, with cognitive dysfunction impairing the ability to allocate attention between cognitive and motor tasks, contributing to gait instability and balance disorders. Age-related nervous system lesions (e.g., white matter lesions) and cardiovascular disease (e.g., heart failure) also increase the likelihood of postural dizziness. As individuals age, the balance system, including semicircular canals and otolith organs, becomes less responsive, contributing to OD in PPPD (87).

#### 3.1.2 Anxiety state and neurotic personality

Approximately three-quarters of PPPD patients experience anxiety (84). Central vestibular pathways and anxiety-related neural networks overlap in the brain. Structural and functional abnormalities in these networks, occurring in patients with mood disorders, may affect vestibular pathways and disrupt postural control (88). Functional MRI studies have shown abnormal connectivity between the prefrontal cortex and visual and motor areas in PPPD patients, with increased activity and connectivity in these regions during vestibular stimulation. This stimulation induces anxiety, postural control issues, and balance dysfunction (89). Indovina et al. (90) found hypofunction and altered connectivity between precortical structures and the parieto-insular vestibular cortex (PIVC), which plays a role in anxiety regulation. This altered connectivity may hinder the return of high-risk postural control to a normal, low-threat strategy. Furthermore, central sensitization is thought to contribute to functional disorders. Anxiety and depression can lead to central sensitization of vestibular sensory pathways under chronic stress, resulting in inappropriate processing of sensory information and contributing to OD in PPPD (91). The interaction of visual-vestibular, sensorimotor, and emotional networks in PPPD patients forms the anatomical basis for increased sensitivity to dizziness during postural and body movement changes. Anxiety states affect motor postural control and spatial orientation, causing heightened sensitivity to somatic posture and movement. This reduces the threshold for adopting high-risk strategies during postural challenges and triggers transitions to high-risk postural control (92). Current drug treatments for PPPD, such as selective serotonin reuptake inhibitors (SSRIs) and serotonin-norepinephrine reuptake inhibitors (SNRIs), target the anxiety circuit and respond to vestibular central neurons, improving anxiety and balance disorders. This confirms the association between anxiety and OD in PPPD.

Neuroticism, a personality trait characterized by excessive worry and negative emotions, is closely associated with psychological dizziness (93). Individuals with confident and optimistic personalities are less likely to experience persistent dizziness after acute vestibular disorders. Neuroticism influences spatial localization and vestibular information processing (94) in PPPD, with mechanisms involving increased responsiveness in the vestibular cortex and subcortical vestibular regions, as well as enhanced connectivity between the vestibular and anxiety systems. Neuroticism affects the visual-vestibular-anxiety system, which in turn influences postural control under stress. Functional MRI studies have confirmed that individuals with a neurotic personality show increased brain network sensitivity in postural control, with hyperactivity in the prefrontal cortex and greater functional connectivity with the motor, premotor, and superior parietal cortex (95). Neuroticism is a risk factor for postural instability in vestibular disease and a predisposing factor for anxiety, with heightened vigilance toward balance (96) potentially contributing to OD in PPPD.

#### 3.1.3 Comorbid vestibular disorders

PPPD can develop following an acute or episodic vestibular disorder. Studies have shown that the chronicity of vestibular neuritis (VN) is significantly increased, with 25%–50% of VN patients unable to fully recover within 3–12 months, ultimately transitioning to PPPD, which exacerbates dizziness during head and physical movements (97). Additionally, vestibular migraine (VM) is one of the most common causes of PPPD, accounting for 49% of cases (98). VM is known for its complex clinical manifestations, often referred to as the “chameleon” of vestibular diseases. Symptoms of VM are typically paroxysmal but can include positional dizziness and persistent postural symptoms. In some cases, VM becomes chronic, which can easily be confused with PPPD. The balance-migraine-anxiety syndrome proposed by Balaban et al. (99), which highlights the overlap between vestibular and pain pathways, involves the imbalance of multiple neural conduction networks, further impacting balance function. Some studies suggest that VM may cause balance disorders through vestibular nucleus dysfunction, leading to self-motion perceptual sensitization (100). The pathogenesis of VM is associated with hyperexcitability in central regions and activation of peripheral vestibular organs. Both the chronic phases of VM and VN can result in excessive sensitivity and vulnerability of central and peripheral nerves, mediating changes in postural control and overlapping with the pathogenesis of PPPD, which likely contributes to OD in PPPD.

### 3.2 The pathogenesis of OD in PPPD

#### 3.2.1 The altered activity and connectivity of cerebral cortical networks

The altered activity and connectivity of cortical networks involved in spatial orientation and threat assessment may contribute to OD in PPPD (101). The PIVC is a core area in the vestibular cortex that processes vestibular input, self-motion perception, and verticality estimation, as well as visual motion, particularly gravity-related motion. A study suggested that reduced activation of the PIVC, hippocampus, anterior insula, inferior frontal gyrus, and anterior cingulate cortex, along with altered connectivity between these regions, may be associated with long-term vestibular symptoms in PPPD patients (90). Some researchers have proposed that the reduced connectivity between the vestibular cortex, frontal regulatory regions, and the hippocampus may underlie the impaired spatial orientation and self-motion perception in PPPD patients (102). Functional MRI (fMRI) has revealed abnormal activation and connectivity in PPPD and related conditions, particularly in the PIVC, visual cortex, cerebellum, and anxiety-related networks (103). In PPPD patients, activity and connectivity between key cortical regions that process spatial motor information (e.g., the posterior insula and hippocampus) and those that modulate threat responses (e.g., the anterior cingulate cortex) were reduced. Changes in the PIVC and hippocampal function impair their ability to assess the relevance of sensory input, whereas inactive and poorly interactive cortical networks fail to suppress the bottom-up influence of threat on instinctive postural control and spatial orientation. This leads to the continued use of high-risk strategies in postural control, making lower levels of posture and gaze control poorly integrated. Studies have also shown that patients with PPPD exhibit altered cortical regulation of visual, vestibular, and sensory input, resulting in a “sensory weighted” disorder (104). This leads to reduced tolerance for perceived instability and deviations in movement perception. Consequently, errors in self-motion prediction and the adoption of high-risk postural control strategies occur, contributing to OD.

Alternatively, diminished functional connectivity between the precuneus and precentral gyrus may be associated with increased symptoms during upright posture and movement in PPPD. Some studies have found that PPPD patients show weakened functional connectivity between the precuneus and precentral gyrus, reducing the ability to use vestibular and visual information to regulate body movement and posture, prompting patients to adopt high-risk postural control strategies to maintain balance (105). Therefore, patients with PPPD exhibit altered cortical network activity and connectivity related to movement control and spatial orientation (106). Brain regions responsible for spatial orientation, multisensory integration, and threat assessment may show reduced activity or connectivity compared to healthy individuals. These changes result in impaired movement control (e.g., upright posture) and abnormal self-motion sensations, such as dizziness.

#### 3.2.2 Autonomic nerve dysfunction

Autonomic dysfunction is prevalent in patients with chronic persistent dizziness. Studies have shown that approximately 80% of patients with chronic persistent dizziness of unknown origin exhibit at least one autonomic abnormality (107). Autonomic dysfunction is also common in PPPD patients, who often experience symptoms such as palpitations, sweating, elevated blood pressure, nausea, anxiety, and fear during episodes of dizziness. Both internal and external stimuli and stress can lead to autonomic imbalance. The autonomic nervous system regulates emotional behavior, cognitive function, and cardiovascular activity, including blood pressure and heart rate, and plays a crucial role in the occurrence of OD in PPPD.

##### 3.2.2.1 Vestibular-autonomic dysfunction and sensory-perceptual dysfunction

Vestibular-autonomic reflex dysfunction and mood disorders are common in patients with OD. The vestibular-autonomic reflex, mediated by vestibular stimulation, activates both sympathetic and parasympathetic pathways, affecting various sympathetic and parasympathetic functions (108). Perception involves the integration of afferent sensory information and internal expectations, and when there is a mismatch between the expected and actual sensory input, persistent dizziness symptoms can arise. The vestibular sensory system and the autonomic nervous system are highly interconnected in the insular cortex and anterior cingulate cortex. The insular cortex plays a key role in interoceptive sensory integration and participates in vestibular and autonomic input and output, whereas the cingulate cortex is crucial for processing emotions like depression and anxiety. Abnormalities in the function of both the insular and cingulate cortices can mediate the perceptual dysfunction of the vestibular-autonomic system and affect central postural control. Imaging studies have shown that anxiety disorder patients exhibit sensory dysfunction and abnormal insular function (109). One study showed that vagus nerve stimulation in PPPD patients significantly improved posture, likely by inducing the release of neurotransmitters that regulate the central cortices (cingulate and insular cortices), positively affecting postural control (110). Autonomic dysfunction can mediate anxiety and depression, which in turn contribute to sensory dysfunction, leading to OD in PPPD. Studies have found that anxiety and sleep disorders are closely associated with autonomic dysfunction. PPPD patients often experience anxiety, sleep disorders, and OD as symptoms of autonomic abnormalities (111). Anxiety and depression are associated with structural, functional, and connectivity changes in brain regions involved in emotional processing, self-reward, and the perception of external stimuli, including networks composed of the hippocampus, amygdala, anterior cingulate cortex, and medial prefrontal cortex. Research has shown that vagal nerve stimulation can improve the abnormal connectivity of these neural networks in depressed patients, leading to improved postural balance control (112). This highlights the close association between the autonomic nervous system and vestibular and cerebral neural networks. PPPD often occurs after acute vestibular events, and vestibular-autonomic dysfunction may be a key mechanism behind OD in PPPD.

##### 3.2.2.2 Vestibular-autonomic dysfunction and hemodynamic changes

Autonomic dysfunction can affect hemodynamics, leading to reduced CBF and OD, which may overlap with diagnostic criteria for OH or POTS (113). When moving from a sitting to a standing position, the body compensates for changes in blood pressure by increasing sympathetic activity, which raises vascular tension, heart contraction, and heart rate to stabilize blood pressure. The maintenance of CBF also relies on sympathetic nerve fibers mediating peripheral vascular contraction and the brain’s own regulatory ability. Without rapid autonomic regulation, individuals are prone to OD in the upright position. The vestibular system is an important regulator of autonomic function and plays a significant role in blood pressure regulation during postural changes. The vestibular-cardiovascular reflex, a component of the vestibular-autonomic reflex, can cause hemodynamic changes when disrupted. The vestibular nerve is closely associated with the cardiovascular system through the medulla’s cardiovascular control center. Clinical studies have shown that stimulation of the vestibular otolith organs can induce peripheral vasoconstriction (114), and during postural shifts from supine to upright, vestibular activation helps stimulate cardiovascular sympathetic nerves, maintaining blood pressure stability. The vestibular-cardiovascular reflex is critical in this process. Studies have shown that when the vestibular nerve is cut, blood pressure fluctuations increase significantly (115), highlighting the role of vestibular stimulation in regulating cardiovascular responses during exercise and postural changes (116). In fact, the vestibular system is involved in controlling blood pressure (117) as part of the autonomic regulation during body movement and postural shifts. Vestibular dysfunction can impair the stability of the cardiovascular baroreceptor reflex, reducing upright endurance in some individuals and contributing to OD. The development of OD due to autonomic dysfunction is primarily associated with widespread hypoperfusion in the cerebral cortex. A study (118) also reported that vertigo regressed after treating autonomic dysfunction. Autonomic nerve-related treatments have been shown to improve dizziness symptoms. As autonomic dysfunction is common in PPPD patients, the hemodynamic changes mediated by this dysfunction may contribute to OD in PPPD.

#### 3.2.3 Changes in postural control

PPPD patients often adopt high-risk postural control strategies, focusing dynamic attention on head and body movements. This results in overshooting of posture and the emergence of postural dizziness symptoms. The subjective imbalance and fearful postural dizziness in PPPD are due to anxiety and excessive sensitivity to posture, which leads to the application of inappropriate balance strategies (119). Several studies have shown that PPPD is associated with high-frequency, low-amplitude postural sway associated with calf muscle co-contraction (58, 120). In a study of chronic subjective dizziness (CSD) patients, similar results were found (59). Although healthy individuals adopt such strategies only under challenging balance conditions, PPPD patients apply these high-risk strategies even in less demanding situations, possibly due to misunderstandings of body movement and excessive sensitivity to positional changes (121). OD in PPPD may be related to a lower threshold for engaging in the closed-loop feedback mechanism that adjusts posture. Postural control is typically governed by a closed-loop mechanism, relying on sensory feedback from the visual, vestibular, and proprioceptive systems to correct deviations from ideal posture caused by gravity, postural changes, or muscle tone fluctuations. A reduced threshold for postural control feedback can lead to the adoption of high-risk strategies unnecessarily, thus diminishing effective postural control. Subjective imbalance is associated with changes in the coordination of open-loop and closed-loop mechanisms in postural control (122). This imbalance further intensifies conscious control of posture, creating a vicious cycle that contributes to OD. Additionally, pathological postural strategies may arise from anticipatory contractions of antigravity muscles due to anxiety (123). As a result, patients with PPPD experience difficulties in postural control across various sensory challenges, and altered postural control may be one of the mechanisms causing OD in PPPD (124).

#### 3.2.4 Otolith dysfunction

Isolated otolith dysfunction (IOD), a newly recognized vestibular condition (125), refers to dysfunction of the saccule and utricle (the otolith organs) whereas maintaining normal semicircular canal function. About 20%–46% of PPPD patients show IOD (126). Chronic otolith dysfunction is closely associated with the development of PPPD. The otolith apparatus has two stimulus response systems: the instantaneous dynamic system and the continuous static system (127), both of which are critical for detecting linear acceleration and head movement, as well as maintaining balance. When the saccule and utricle are dysfunctional, the body experiences abnormal perception of horizontal and vertical accelerations, leading to symptoms of OD. Vestibular examinations in PPPD patients have found that most patients exhibit otolith dysfunction, with many showing IOD (128). Moreover, otolith dysfunction can reduce the vestibular-sympathetic reflex, contributing to OD. Studies involving linear acceleration movements have demonstrated that vestibular regulation of autonomic activity plays a role in blood pressure regulation (129). Another study on otolith disorders and cardiovascular autonomic function (130) found a higher incidence of OH during active standing in patients with otolith dysfunction compared to those with normal cervical vestibular-induced myogenic potentials. This suggests that otolith dysfunction may induce OH. Given its role in sensing linear acceleration and mediating the vestibular-sympathetic reflex, dysfunction of the otolith organs may contribute to increased OD in PPPD patients.

#### 3.2.5 Visual and somatosensory dependence

PPPD patients primarily rely on visual input rather than vestibular input for spatial orientation, leading to altered sensory integration that favors visual or somatosensory cues (131). During acute vestibular syndromes, patients may depend on visual and somatosensory inputs to control posture. However, in PPPD patients, this physiological shift in sensory reliance does not return to normal. As a result, when upright or walking, patients excessively depend on vision to perceive their external environment, and visual stimulation combined with vestibular dysfunction leads to “sensory weighted” disorder. These disorders cause abnormal postural control. Functional MRI (fMRI) studies in PPPD patients show enhanced functional connectivity between the sensorimotor network (including the motor cortex, sensorimotor cortex, and secondary somatosensory cortex) and the occipital visual network (94). These patients also exhibit increased activity in the visual cortex and reduced connectivity with the precentral gyrus (the primary motor center). Neuroimaging studies in PPPD further support the role of visual dependency in influencing motor control and spatial orientation. Sensory organization tests (SOT) in PPPD patients revealed that 45% showed abnormal sensory processing, with a marked tendency towards visual or somatosensory dependence, which worsens postural control (132). It has been suggested that in standing posture, PPPD patients rely more on visual input and less on somatosensory input than healthy individuals (133). This aligns with clinical observations, where vision significantly influences standing posture control in PPPD patients (134). Combined with their dependence on somatosensory input, this explains the patients’ heightened visual sensitivity and reliance. It also clarifies why standing dizziness decreases when patients touch an object, highlighting a key mechanism underlying OD in PPPD.

#### 3.2.6 Neurotransmitter abnormalities

Serotonin (5-HT) and norepinephrine (NE) play crucial roles in regulating the activities of central and peripheral neurons, contributing to the central balance control pathways. These neurotransmitters may be involved in the postural control mechanisms of PPPD. In an experiment involving repetitive brain injury in male rats (135), balance was significantly impaired, with NE levels significantly increased in the locus coeruleus, decreased in the vestibular nucleus, and 5-HT levels increased in the motor cortex. This suggests that dysregulation of NE and 5-HT in areas related to motor control may contribute to balance disorders. The brainstem vestibular nucleus is vital for posture and balance control. The inferior vestibular nucleus (IVN), the largest nucleus in the vestibular complex, plays a significant role in integrating information signals that control body posture. NE induces excitatory responses in neurons of the IVN. A study on CSD patients found abnormalities in both NE and 5-HT levels in their serum (136). Extracellular recordings of rat brainstem slices and quantitative real-time RT-PCR studies revealed that NE directly regulates the activity of IVN neurons through α1-, α2-, and β2-receptors, indicating that the central noradrenergic system may actively participate in IVN-mediated vestibular reflexes and postural control (137). Serotonin receptors are found throughout the vestibular pathway in the brain, with spirochetes and vestibular ganglion cells expressing 5-HT receptors. In the CNS, 5-HT neurons are mainly distributed in the raphe nuclei, with dense projections throughout the brain. When 5-HT is released, it binds to 5-HT receptors, which then regulate brain functions. Some studies have shown that square dancing combined with SSRIs has a positive effect on dizziness and balance in middle-aged and elderly women with PPPD (138). This suggests that dysregulation of NE and 5-HT levels in motor-related brain regions may contribute to balance disorders. Current pharmacological treatments for PPPD, primarily SSRIs and SNRIs (3), may work by reducing excessive excitability or improving psychological symptoms (such as anxiety) commonly seen in PPPD patients. These medications may also directly influence the extensive balance network in the brain, suggesting a role in regulating balance.

## 4 Summary

In conclusion, management of OD associated with PPPD, attention should be paid not only to differential diagnosis but also to the presence of PPPD comorbidities with many chronic dizziness disorders. The present review also emphasizes the importance of considering the fact that OD is not necessarily associated with orthostatic hypotension. Given the growing evidence of the extensive mechanisms between the vestibular system and various OD relevant diseases, a comprehensive approach to diagnosis and treatment is essential for PPPD patients. OD in PPPD is a complex process influenced by multiple factors and mechanisms. Ongoing research aims to better understand the mechanisms and identify diagnostic biomarkers for OD in PPPD, enabling early intervention and preventing adverse effects on workability. For patients with established PPPD, it is important to identify the etiologies of OD, track progress, and evaluate responses to therapies to reduce diagnostic errors and missed diagnoses. When treating OD in PPPD, it is recommended to consider multimodal integration rather than focusing solely on posturography deficits or imaging abnormalities.

## References

[1] Kim HJ Lee JO Choi JY Kim JS. Etiologic distribution of dizziness and vertigo in a referral-based dizziness clinic in South Korea. J Neurol. (2020) 267:2252–9. doi: 10.1007/s00415-020-09831-2, PMID: 32300888

[2] Habs M Strobl R Grill E Dieterich M Becker-Bense S. Primary or secondary chronic functional dizziness: does it make a difference? A DizzyReg study in 356 patients. J Neurol. (2020) 267:212–22. doi: 10.1007/s00415-020-10150-9, PMID: 32852579 PMC7718176

[3] Hüfner K Sperner-Unterweger B. Persistent-postural perceptual dizziness (PPPD): yes, it is a psychosomatic condition! J Vestib Res. (2023) 33:279–81. doi: 10.3233/VES-190679, PMID: 31561401

[4] Popkirov S Staab JP Stone J. Persistent postural-perceptual dizziness (PPPD): a common, characteristic and treatable cause of chronic dizziness. Pract Neurol. (2018) 18:5–13. doi: 10.1136/practneurol-2017-001809, PMID: 29208729

[5] Woll J Sprenger A Helmchen C. Postural control during galvanic vestibular stimulation in patients with persistent perceptual-postural dizziness. J Neurol. (2019) 266:1236–49. doi: 10.1007/s00415-019-09255-7, PMID: 30809703

[6] Radtke A Lempert T von Brevern M Feldmann M Lezius F Neuhauser H. Prevalence and complications of orthostatic dizziness in the general population. Clin Auton Res. (2011) 21:161–8. doi: 10.1007/s10286-010-0114-2, PMID: 21279415

[7] Claffey P Pérez-Denia L Lavan A Kenny RA Finucane C Briggs R. Asymptomatic orthostatic hypotension and risk of falls in community-dwelling older people. Age Ageing. (2022) 51:afac295. doi: 10.1093/ageing/afac295, PMID: 36571778

[8] Wieling W Kaufmann H Claydon VE van Wijnen VK Harms MPM Juraschek SP. Diagnosis and treatment of orthostatic hypotension. Lancet Neurol. (2022) 21:735–46. doi: 10.1016/S1474-4422(22)00169-7, PMID: 35841911 PMC10024337

[9] Bogle JM Benarroch E Sandroni P. Vestibular-autonomic interactions: beyond orthostatic dizziness. Curr Opin Neurol. (2022) 35:126–34. doi: 10.1097/WCO.0000000000001013, PMID: 34839339

[10] Staab JP Eckhardt-Henn A Horii A Jacob R Strupp M Brandt T. Diagnostic criteria for persistent postural-perceptual dizziness (PPPD): consensus document of the committee for the classification of vestibular disorders of the Bárány society. J Vestib Res. (2017) 27:191–208. doi: 10.3233/VES-170622, PMID: 29036855 PMC9249299

[11] Juraschek SP Longstreth WT Jr Lopez OL Gottdiener JS Lipsitz LA Kuller LH. Orthostatic hypotension, dizziness, neurology outcomes, and death in older adults. Neurology. (2020) 95:e1941–50. doi: 10.1212/WNL.0000000000010456, PMID: 32732296 PMC7682840

[12] Kim HA Bisdorff A Bronstein AM Lempert T Rossi-Izquierdo M Staab JP. Hemodynamic orthostatic dizziness/vertigo: diagnostic criteria. J Vestib Res. (2019) 29:45–56. doi: 10.3233/VES-190655, PMID: 30883381 PMC9249281

[13] Kim HA Yi HA Lee H. Spectrum of autonomic dysfunction in orthostatic dizziness. Clin Neurophysiol. (2014) 125:1248–54. doi: 10.1016/j.clinph.2013.10.022, PMID: 24268815

[14] Thieben MJ Sandroni P Sletten DM Benrud-Larson LM Fealey RD Vernino S. Postural orthostatic tachycardia syndrome: the Mayo Clinic experience. Mayo Clin Proc. (2007) 82:308–13. doi: 10.4065/82.3.308, PMID: 17352367

[15] Mar PL Raj SR. Postural orthostatic tachycardia syndrome: mechanisms and new therapies. Annu Rev Med. (2020) 71:235–48. doi: 10.1146/annurev-med-041818-011630, PMID: 31412221

[16] Olshansky B Cannom D Fedorowski A Stewart J Gibbons C Sutton R. Postural orthostatic tachycardia syndrome (POTS): a critical assessment. Prog Cardiovasc Dis. (2020) 63:263–70. doi: 10.1016/j.pcad.2020.03.010, PMID: 32222376 PMC9012474

[17] Fedorowski A. Postural orthostatic tachycardia syndrome: clinical presentation, aetiology and management. J Intern Med. (2019) 285:352–66. doi: 10.1111/joim.12852, PMID: 30372565

[18] Angeli AM Salonen BR Ganesh R Hurt RT Abdalrhim A Mueller M. Symptom presentation by phenotype of postural orthostatic tachycardia syndrome. Sci Rep. (2024) 14:205. doi: 10.1038/s41598-023-50886-8, PMID: 38168762 PMC10761725

[19] Kharraziha I Holm H Bachus E Ricci F Sutton R Fedorowski A. Cerebral oximetry in syncope and syndromes of orthostatic intolerance. Front Cardiovasc Med. (2019) 6:171. doi: 10.3389/fcvm.2019.00171, PMID: 31824964 PMC6886369

[20] Shaw BH Stiles LE Bourne K Green EA Shibao CA Okamoto LE. The face of postural tachycardia syndrome – insights from a large cross-sectional online community-based survey. J Intern Med. (2019) 286:438–48. doi: 10.1111/joim.12895, PMID: 30861229 PMC6790699

[21] Blitshteyn S. Is postural orthostatic tachycardia syndrome (POTS) a central nervous system disorder? J Neurol. (2022) 269:725–32. doi: 10.1007/s00415-021-10502-z, PMID: 33677650 PMC7936931

[22] Kwon H Kwon E Kim HJ Choi JY Kim JS. Vestibular syncope: clinical characteristics and mechanism. Ann Clin Transl Neurol. (2022) 9:1616–25. doi: 10.1002/acn3.51661, PMID: 36056529 PMC9539380

[23] Kwon E Lee JY Kim HJ Choi JY Kim JS. Can dyssynergia of vestibulosympathetic and baroreflexes cause vestibular syncope? The hypothesis based on the velocity-storage function. Cerebellum. (2022) 21:244–52. doi: 10.1007/s12311-021-01296-x, PMID: 34156636

[24] Choi JY Koo YJ Song JM Kim HJ Kim JS. Effect of a false inertial cue in the velocity-storage circuit on head posture and inertia perception. J Neurosci. (2023) 43:1530–9. doi: 10.1523/JNEUROSCI.1148-22.2023, PMID: 36669887 PMC10008054

[25] Lackner JR DiZio P. Velocity storage: its multiple roles. J Neurophysiol. (2020) 123:1206–15. doi: 10.1152/jn.00139.2019, PMID: 31913743

[26] Imai T Takeda N Ikezono T Shigeno K Asai M Watanabe Y. Classification, diagnostic criteria and management of benign paroxysmal positional vertigo. Auris Nasus Larynx. (2017) 44:1–6. doi: 10.1016/j.anl.2016.03.013, PMID: 27174206

[27] Kim MJ Rhim GI. Relationship between orthostatic hypotension and recurrence of benign paroxysmal positional vertigo. Sci Rep. (2022) 12:10685. doi: 10.1038/s41598-022-15029-5, PMID: 35739188 PMC9226118

[28] Jeon EJ Park YS Park SN Park KH Kim DH Nam IC. Clinical significance of orthostatic dizziness in the diagnosis of benign paroxysmal positional vertigo and orthostatic intolerance. Am J Otolaryngol. (2013) 34:471–6. doi: 10.1016/j.amjoto.2013.04.005, PMID: 23790615

[29] Pezzoli M Garzaro M Pecorari G Cena M Giordano C Albera R. Benign paroxysmal positional vertigo and orthostatic hypotension. Clin Auton Res. (2010) 20:27–31. doi: 10.1007/s10286-009-0032-3, PMID: 19820989

[30] Kim HA Lee H. Autonomic dysfunction as a possible cause of residual dizziness after successful treatment in benign paroxysmal positional vertigo. Clin Neurophysiol. (2014) 125:608–14. doi: 10.1016/j.clinph.2013.08.008, PMID: 24045026

[31] First MB Clarke DE Yousif L Eng AM Gogtay N Appelbaum PS. DSM-5-TR: rationale, process, and overview of changes. Psychiatr Serv. (2023) 74:869–75. doi: 10.1176/appi.ps.20220334, PMID: 36510761

[32] Staab JP Ruckenstein MJ. Autonomic nervous system function in chronic dizziness. Otol Neurotol. (2007) 28:854–9. doi: 10.1097/MAO.0b013e31805c74a7, PMID: 17514065

[33] Maslovara S Begic D Butkovic-Soldo S Vceva A Pajic-Matic I Sestak A. Are the persistent postural-perceptual dizziness (PPPD) patients more anxious than the patients with other dizziness? Psychiatr Danub. (2022) 34:71–8. doi: 10.24869/psyd.2022.71 35467613

[34] Huppert D Wuehr M Brandt T. Acrophobia and visual height intolerance: advances in epidemiology and mechanisms. J Neurol. (2020) 267:231–40. doi: 10.1007/s00415-020-09805-4, PMID: 32444982 PMC7718183

[35] Lucieer F Vonk P Guinand N Stokroos R Kingma H van de Berg R. Bilateral vestibular hypofunction: insights in etiologies, clinical subtypes, and diagnostics. Front Neurol. (2016) 7:26. doi: 10.3389/fneur.2016.00026, PMID: 26973594 PMC4777732

[36] Strupp M Kim JS Murofushi T Straumann D Jen JC Rosengren SM. Bilateral vestibulopathy: diagnostic criteria consensus document of the classification committee of the Bárány society. J Vestib Res. (2017) 27:177–89. doi: 10.3233/VES-170619, PMID: 29081426 PMC9249284

[37] Starkov D Strupp M Pleshkov M Kingma H van de Berg R. Diagnosing vestibular hypofunction: an update. J Neurol. (2021) 268:377–85. doi: 10.1007/s00415-020-10139-4, PMID: 32767115 PMC7815536

[38] Hannigan IP Welgampola MS Watson SRD. Dissociation of caloric and head impulse tests: a marker of Meniere's disease. J Neurol. (2021) 268:431–9. doi: 10.1007/s00415-019-09431-9, PMID: 31222419

[39] Tamanini JB Mezzalira R Vallim MGB Gabriel GP Stoler G Chone CT. Dissociation between video head impulse test and caloric test: a marker of Menière's disease? – a systematic review and meta-analysis. Braz J Otorhinolaryngol. (2023) 89:101279. doi: 10.1016/j.bjorl.2023.101279, PMID: 37354884 PMC10331280

[40] Kim JS Kim HJ. Bilateral vestibulopathy: the causes, diagnosis, and treatments. Curr Opin Neurol. (2022) 35:98–106. doi: 10.1097/WCO.0000000000001014, PMID: 34879018

[41] Ishai R Seyyedi M Chancellor AM McLean CA Rodriguez ML Halmagyi GM. The pathology of the vestibular system in CANVAS. Otol Neurotol. (2021) 42:e332–40. doi: 10.1097/MAO.0000000000002985, PMID: 33492056 PMC9234914

[42] Lenka A Jankovic J. Tremor syndromes: an updated review. Front Neurol. (2021) 12:684835. doi: 10.3389/fneur.2021.684835, PMID: 34381412 PMC8350038

[43] Hassan A Ahlskog JE Matsumoto JY Milber JM Bower JH Wilkinson JR. Orthostatic tremor: clinical, electrophysiologic, and treatment findings in 184 patients. Neurology. (2016) 86:458–64. doi: 10.1212/WNL.0000000000002328 26747880

[44] Karlberg M Fransson PA Magnusson M. Posturography can be used to screen for primary orthostatic tremor, a rare cause of dizziness. Otol Neurotol. (2005) 26:1200–3. doi: 10.1097/01.mao.0000194891.26097.e0, PMID: 16272942

[45] Swinnen BEKS de Waal H Buijink AWG de Bie RMA van Rootselaar AF. The phenomenology of primary orthostatic tremor. Mov Disord Clin Pract. (2022) 9:489–93. doi: 10.1002/mdc3.13454, PMID: 35582311 PMC9092733

[46] Benito-León J Domingo-Santos Á. Orthostatic tremor: an update on a rare entity. Tremor Other Hyperkinet Mov (N Y). (2016) 6:411. doi: 10.7916/D81N81BT, PMID: 27713855 PMC5039949

[47] Lenka A Pal PK Bhatti DE Louis ED. Pathogenesis of primary orthostatic tremor: current concepts and controversies. Tremor Other Hyperkinet Mov (N Y). (2017) 7:513. doi: 10.7916/D8W66ZBH, PMID: 29204315 PMC5712672

[48] Schöberl F Feil K Xiong G Bartenstein P la Fougére C Jahn K. Pathological ponto-cerebello-thalamo-cortical activations in primary orthostatic tremor during lying and stance. Brain. (2017) 140:83–97. doi: 10.1093/brain/aww268, PMID: 28031220

[49] Thomson FJ Masson EA Boulton AJ. The clinical diagnosis of sensory neuropathy in elderly people. Diabet Med. (1993) 10:843–6. doi: 10.1111/j.1464-5491.1993.tb00177.x, PMID: 8281730

[50] Gwathmey KG. Sensory polyneuropathies. Continuum (Minneap Minn). (2017) 23:1411–36. doi: 10.1212/CON.0000000000000518, PMID: 28968369

[51] Samaha M Katsarkas A. Vestibular impairment in peripheral sensory neuropathies. J Otolaryngol. (2000) 29:299–301. 11108489

[52] Jáuregui-Renaud K Aranda-Moreno C Herrera-Rangel A. Utricular hypofunction in patients with type 2 diabetes mellitus. Acta Otorhinolaryngol Ital. (2017) 37:430–5. doi: 10.14639/0392-100X-1243, PMID: 28530263 PMC5717987

[53] Raccagni C Nonnekes J Bloem BR Peball M Boehme C Seppi K. Gait and postural disorders in Parkinsonism: a clinical approach. J Neurol. (2020) 267:3169–76. doi: 10.1007/s00415-019-09382-1, PMID: 31119450 PMC7578144

[54] Kwon KY You J Kim RO Lee EJ. Association of dizziness-related handicap or disability with clinical features in patients with early Parkinson's disease. J Integr Neurosci. (2023) 22:68. doi: 10.31083/j.jin2203068, PMID: 37258439

[55] Kim JG Kim SH Lee SU Lee CN Kim BJ Kim JS. Head-impulse tests aid in differentiation of multiple system atrophy from Parkinson's disease. J Neurol. (2022) 269:2972–9. doi: 10.1007/s00415-021-10885-z, PMID: 34767067

[56] Scarpa A Cassandro C Vitale C Ralli M Policastro A Barone P. A comparison of auditory and vestibular dysfunction in Parkinson's disease and multiple system atrophy. Parkinsonism Relat Disord. (2020) 71:51–7. doi: 10.1016/j.parkreldis.2020.01.018, PMID: 32032926

[57] Cerchiai N Mancuso M Navari E Giannini N Casani AP. Aging with cerebral small vessel disease and dizziness: the importance of undiagnosed peripheral vestibular disorders. Front Neurol. (2017) 8:241. doi: 10.3389/fneur.2017.00241, PMID: 28626444 PMC5454069

[58] Wuehr M Pradhan C Novozhilov S Krafczyk S Brandt T Jahn K. Inadequate interaction between open- and closed-loop postural control in phobic postural vertigo. J Neurol. (2013) 260:1314–23. doi: 10.1007/s00415-012-6797-7, PMID: 23263595

[59] Dieterich M Staab JP. Functional dizziness: from phobic postural vertigo and chronic subjective dizziness to persistent postural-perceptual dizziness. Curr Opin Neurol. (2017) 30:107–13. doi: 10.1097/WCO.0000000000000417, PMID: 28002123

[60] Ahmad H Cerchiai N Mancuso M Casani AP Bronstein AM. Are white matter abnormalities associated with "unexplained dizziness"? J Neurol Sci. (2015) 358:428–31. doi: 10.1016/j.jns.2015.09.006, PMID: 26412160 PMC4640145

[61] Kaski D Rust HM Ibitoye R Arshad Q Allum JHJ Bronstein AM. Theoretical framework for "unexplained" dizziness in the elderly: the role of small vessel disease. Prog Brain Res. (2019) 248:225–40. doi: 10.1016/bs.pbr.2019.04.009, PMID: 31239134

[62] Ulivi L Maccarrone M Giannini N Ferrari E Caselli MC Montano V. Oxidative stress in cerebral small vessel disease dizziness patients, basally and after polyphenol compound supplementation. Curr Mol Med. (2018) 18:160–5. doi: 10.2174/1566524018666180720165055, PMID: 30033867 PMC6225324

[63] Newman-Toker DE Dy FJ Stanton VA Zee DS Calkins H Robinson KA. How often is dizziness from primary cardiovascular disease true vertigo? A systematic review. J Gen Intern Med. (2008) 23:2087–94. doi: 10.1007/s11606-008-0801-z, PMID: 18843523 PMC2596492

[64] Culić V Mirić D Eterović D. Correlation between symptomatology and site of acute myocardial infarction. Int J Cardiol. (2001) 77:163–8. doi: 10.1016/s0167-5273(00)00414-9, PMID: 11182180

[65] Goldberger ZD Petek BJ Brignole M Shen WK Sheldon RS Solbiati M. ACC/AHA/HRS versus ESC guidelines for the diagnosis and management of syncope: JACC guideline comparison. J Am Coll Cardiol. (2019) 74:2410–23. doi: 10.1016/j.jacc.2019.09.012, PMID: 31699282

[66] Kim HA Ahn J Park HS Lee SM Choi SY Oh EH. Cardiogenic vertigo: characteristics and proposed diagnostic criteria. J Neurol. (2021) 268:1070–5. doi: 10.1007/s00415-020-10252-4, PMID: 33025120

[67] Jolobe O. Potential causes of delayed diagnosis include convulsive syncope and cardiogenic vertigo. QJM. (2010) 103:59. doi: 10.1093/qjmed/hcp112, PMID: 19671669

[68] Choi JH Yang TI Cha SY Lee TH Choi KD Kim JS. Ictal downbeat nystagmus in cardiogenic vertigo. Neurology. (2010) 75:2129–30. doi: 10.1212/WNL.0b013e318200d752, PMID: 21135388

[69] Tran TM Lee MS McClelland CM. Downbeat nystagmus: a clinical review of diagnosis and management. Curr Opin Ophthalmol. (2021) 32:504–14. doi: 10.1097/ICU.0000000000000802, PMID: 34456290

[70] Claassen JAHR Thijssen DHJ Panerai RB Faraci FM. Regulation of cerebral blood flow in humans: physiology and clinical implications of autoregulation. Physiol Rev. (2021) 101:1487–559. doi: 10.1152/physrev.00022.2020, PMID: 33769101 PMC8576366

[71] Novak P. Orthostatic cerebral hypoperfusion syndrome. Front Aging Neurosci. (2016) 8:22. doi: 10.3389/fnagi.2016.00022, PMID: 26909037 PMC4754393

[72] Cai C Fordsmann JC Jensen SH Gesslein B Lønstrup M Hald BO. Stimulation-induced increases in cerebral blood flow and local capillary vasoconstriction depend on conducted vascular responses. Proc Natl Acad Sci USA. (2018) 115:E5796–804. doi: 10.1073/pnas.1707702115, PMID: 29866853 PMC6016812

[73] Skoog J Lindenberger M Ekman M Holmberg B Zachrisson H Länne T. Reduced venous compliance: an important determinant for orthostatic intolerance in women with vasovagal syncope. Am J Physiol Regul Integr Comp Physiol. (2016) 310:R253–61. doi: 10.1152/ajpregu.00362.2015, PMID: 26561647

[74] Novak P. Post COVID-19 syndrome associated with orthostatic cerebral hypoperfusion syndrome, small fiber neuropathy and benefit of immunotherapy: a case report. eNeurologicalSci. (2020) 21:100276. doi: 10.1016/j.ensci.2020.100276, PMID: 32984564 PMC7502253

[75] Luetzen N Dovi-Akue P Fung C Beck J Urbach H. Spontaneous intracranial hypotension: diagnostic and therapeutic workup. Neuroradiology. (2021) 63:1765–72. doi: 10.1007/s00234-021-02766-z, PMID: 34297176 PMC8528761

[76] Schievink WI. Spontaneous intracranial hypotension. N Engl J Med. (2021) 385:2173–8. doi: 10.1056/NEJMra2101561, PMID: 34874632

[77] Chung SJ Kim JS Lee MC. Syndrome of cerebral spinal fluid hypovolemia: clinical and imaging features and outcome. Neurology. (2000) 55:1321–7. doi: 10.1212/wnl.55.9.1321, PMID: 11087775

[78] Ferrante E Regna-Gladin C Citterio A Arpino I. Spontaneous intracranial hypotension syndrome with hearing loss and pachymeningeal enhancement in the internal acoustic canal: neuroimaging correlations. J Craniofac Surg. (2010) 21:1660–1. doi: 10.1097/SCS.0b013e3181efab0b, PMID: 20856076

[79] Choi JH Cho KY Cha SY Seo JD Kim MJ Choi YR. Audiovestibular impairments associated with intracranial hypotension. J Neurol Sci. (2015) 357:96–100. doi: 10.1016/j.jns.2015.07.002, PMID: 26165775

[80] Xia P Zhang SR Zhou ZJ Shao YQ Hu XY. Benign paroxysmal positional vertigo in spontaneous intracranial hypotension. Neurol Res. (2018) 40:868–73. doi: 10.1080/01616412.2018.1495883, PMID: 30052143

[81] Godemann F Siefert K Hantschke-Brüggemann M Neu P Seidl R Ströhle A. What accounts for vertigo one year after neuritis vestibularis – anxiety or a dysfunctional vestibular organ? J Psychiatr Res. (2005) 39:529–34. doi: 10.1016/j.jpsychires.2004.12.006, PMID: 15992562

[82] Heinrichs N Edler C Eskens S Mielczarek MM Moschner C. Predicting continued dizziness after an acute peripheral vestibular disorder. Psychosom Med. (2007) 69:700–7. doi: 10.1097/PSY.0b013e318151a4dd, PMID: 17766688

[83] Bittar RS Lins EM. Clinical characteristics of patients with persistent postural-perceptual dizziness. Braz J Otorhinolaryngol. (2015) 81:276–82. doi: 10.1016/j.bjorl.2014.08.012, PMID: 25382427 PMC9452260

[84] Park JH Nguyen TT Kim SH Park JY Na S Jeon EJ. Clinical characteristics of persistent postural-perceptual dizziness and its visual subtype in Korean patients: a multicenter cross-sectional study. Brain Behav. (2024) 14:e3389. doi: 10.1002/brb3.3389 38391108 PMC10831130

[85] Wiesmeier IK Dalin D Maurer C. Elderly use proprioception rather than visual and vestibular cues for postural motor control. Front Aging Neurosci. (2015) 7:97. doi: 10.3389/fnagi.2015.00097, PMID: 26157386 PMC4477145

[86] Lamoth CJ van Deudekom FJ van Campen JP Appels BA de Vries OJ Pijnappels M. Gait stability and variability measures show effects of impaired cognition and dual tasking in frail people. J Neuroeng Rehabil. (2011) 8:2. doi: 10.1186/1743-0003-8-2, PMID: 21241487 PMC3034676

[87] De Chua KW Yuen HW Low DAYM Kamath S. Proposal on the diagnostic criteria of definite isolated otolith dysfunction. J Audiol Otol. (2021) 25:59–60. doi: 10.7874/jao.2020.00535, PMID: 33327705 PMC7835430

[88] Ibrahim NMK Hazza NMA Yaseen DM Galal EM. Effect of vestibular rehabilitation games in patients with persistent postural perceptual dizziness and its relation to anxiety and depression: prospective study. Eur Arch Otorrinolaringol. (2024) 281:2861–9. doi: 10.1007/s00405-023-08369-z, PMID: 38127098 PMC11065905

[89] Teh CS Mah MC Rahmat K Prepageran N. Neuroimaging systematic review in persistent postural-perceptual dizziness: the elaborate alterations in the delicate network to remain balanced. Otol Neurotol. (2022) 43:12–22. doi: 10.1097/MAO.0000000000003389, PMID: 34669685

[90] Indovina I Passamonti L Mucci V Chiarella G Lacquaniti F Staab JP. Brain correlates of persistent postural-perceptual dizziness: a review of neuroimaging studies. J Clin Med. (2021) 10:4274. doi: 10.3390/jcm10184274, PMID: 34575385 PMC8468644

[91] Indovina I Riccelli R Staab JP Lacquaniti F Passamonti L. Personality traits modulate subcortical and cortical vestibular and anxiety responses to sound-evoked otolithic receptor stimulation. J Psychosom Res. (2014) 77:391–400. doi: 10.1016/j.jpsychores.2014.09.005, PMID: 25262497

[92] Hashimoto K Takeuchi T Ueno T Suka S Hiiragi M Yamada M. Effect of central sensitization on dizziness-related symptoms of persistent postural-perceptual dizziness. BioPsycho Soc Med. (2022) 16:7. doi: 10.1186/s13030-022-00235-4, PMID: 35255948 PMC8900397

[93] Chiarella G Petrolo C Riccelli R Giofrè L Olivadese G Gioacchini FM. Chronic subjective dizziness: analysis of underlying personality factors. J Vestib Res. (2016) 26:403–8. doi: 10.3233/VES-160590, PMID: 27814314

[94] Yagi C Morita Y Kitazawa M Yamagishi T Ohshima S Izumi S. Subtypes of persistent postural-perceptual dizziness. Front Neurol. (2021) 12:652366. doi: 10.3389/fneur.2021.652366, PMID: 33935950 PMC8085253

[95] Im JJ Na S Jeong H Chung YA. A review of neuroimaging studies in persistent postural-perceptual dizziness (PPPD). Nucl Med Mol Imaging. (2021) 55:53–60. doi: 10.1007/s13139-020-00675-2, PMID: 33968271 PMC8053630

[96] Passamonti L Riccelli R Lacquaniti F Staab JP Indovina I. Brain responses to virtual reality visual motion stimulation are affected by neurotic personality traits in patients with persistent postural-perceptual dizziness. J Vestib Res. (2018) 28:369–78. doi: 10.3233/VES-190653, PMID: 30856138

[97] Oka M Ichijo K Koda K Kamogashira T Kinoshita M Igarashi K. Preceding balance disorders affect vestibular function in persistent postural-perceptual dizziness. J Clin Med. (2023) 12:2589. doi: 10.3390/jcm12072589, PMID: 37048672 PMC10095344

[98] Eggers SDZ Staab JP. Vestibular migraine and persistent postural perceptual dizziness. Handb Clin Neurol. (2024) 199:389–411. doi: 10.1016/B978-0-12-823357-3.00028-8, PMID: 38307659

[99] Balaban CD Jacob RG Furman JM. Neurologic bases for comorbidity of balance disorders, anxiety disorders and migraine: neurotherapeutic implications. Expert Rev Neurother. (2011) 11:379–94. doi: 10.1586/ern.11.19, PMID: 21375443 PMC3107725

[100] King S Priesol AJ Davidi SE Merfeld DM Ehtemam F Lewis RF. Self-motion perception is sensitized in vestibular migraine: pathophysiologic and clinical implications. Sci Rep. (2019) 9:14323. doi: 10.1038/s41598-019-50803-y, PMID: 31586151 PMC6778132

[101] von Söhsten Lins EMD Bittar RSM Bazán PR Amaro Júnior E Staab JP. Cerebral responses to stationary emotional stimuli measured by fMRI in women with persistent postural-perceptual dizziness. Int Arch Otorhinolaryngol. (2021) 25:e355–64. doi: 10.1055/s-0040-1716572, PMID: 34377168 PMC8321645

[102] Castro P Bancroft MJ Arshad Q Kaski D. Persistent postural-perceptual dizziness (PPPD) from brain imaging to behaviour and perception. Brain Sci. (2022) 12:753. doi: 10.3390/brainsci12060753, PMID: 35741638 PMC9220882

[103] Cao Z Liu X Ju Y Zhao X. Neuroimaging studies in persistent postural-perceptual dizziness and related disease: a systematic review. J Neurol. (2022) 269:1225–35. doi: 10.1007/s00415-021-10558-x, PMID: 34019178

[104] Yamaguchi T Miwa T Tamura K Inoue F Umezawa N Maetani T. Temporal virtual reality-guided, dual-task, trunk balance training in a sitting position improves persistent postural-perceptual dizziness: proof of concept. J Neuroeng Rehabil. (2022) 19:92. doi: 10.1186/s12984-022-01068-6, PMID: 35987778 PMC9392908

[105] Li K Si L Cui B Ling X Shen B Yang X. Altered spontaneous functional activity of the right precuneus and cuneus in patients with persistent postural-perceptual dizziness. Brain Imaging Behav. (2020) 14:2176–86. doi: 10.1007/s11682-019-00168-7, PMID: 31313022

[106] Staab JP. Persistent postural-perceptual dizziness. Semin Neurol. (2020) 40:130–7. doi: 10.1055/s-0039-3402736, PMID: 31935771

[107] Lee H Ha K. Autonomic dysfunction in chronic persistent dizziness. J Neurol Sci. (2014) 344:165–70. doi: 10.1016/j.jns.2014.06.048, PMID: 25012479

[108] Raphan T. Vestibular, locomotor, and vestibulo-autonomic research: 50 years of collaboration with Bernard Cohen. J Neurophysiol. (2020) 123:329–45. doi: 10.1152/jn.00485.2019, PMID: 31747361 PMC6985855

[109] Eren OE Filippopulos F Sönmez K Möhwald K Straube A Schöberl F. Non-invasive vagus nerve stimulation significantly improves quality of life in patients with persistent postural-perceptual dizziness. J Neurol. (2018) 265:63–9. doi: 10.1007/s00415-018-8894-8, PMID: 29785522

[110] Grimaldi D Reid KJ Papalambros NA Braun RI Malkani RG Abbott SM. Autonomic dysregulation and sleep homeostasis in insomnia. Sleep. (2021) 44:zsaa274. doi: 10.1093/sleep/zsaa274, PMID: 33295989 PMC8343579

[111] Santana MDR Garner DM de Moraes YM Mangueira LB Alcantara GC da Silva JRA. Association between hospital anxiety sepression scale and autonomic recovery following exercise. J Clin Psychol Med Settings. (2020) 27:295–304. doi: 10.1007/s10880-019-09683-7 31776757

[112] Fang J Rong P Hong Y Fan Y Liu J Wang H. Transcutaneous vagus nerve stimulation modulates default mode network in major depressive disorder. Biol Psychiatry. (2016) 79:266–73. doi: 10.1016/j.biopsych.2015.03.025, PMID: 25963932 PMC4838995

[113] Stewart JM. Common syndromes of orthostatic intolerance. Pediatrics. (2013) 131:968–80. doi: 10.1542/peds.2012-2610, PMID: 23569093 PMC3639459

[114] Kim YH Paik SH V ZP Jeon NJ Kim BJ Kim BM. Cerebral perfusion monitoring using near-infrared spectroscopy during head-up tilt table test in patients with orthostatic intolerance. Front Hum Neurosci. (2019) 13:55. doi: 10.3389/fnhum.2019.00055, PMID: 30837856 PMC6389826

[115] Kaufmann H Biaggioni I Voustianiouk A Diedrich A Costa F Clarke R. Vestibular control of sympathetic activity. An otolith-sympathetic reflex in humans. Exp Brain Res. (2002) 143:463–9. doi: 10.1007/s00221-002-1002-3, PMID: 11914792

[116] Yates BJ Aoki M Burchill P Bronstein AM Gresty MA. Cardiovascular responses elicited by linear acceleration in humans. Exp Brain Res. (1999) 125:476–84. doi: 10.1007/s002210050705, PMID: 10323294

[117] Yates BJ Bronstein AM. The effects of vestibular system lesions on autonomic regulation: observations, mechanisms, and clinical implications. J Vestib Res. (2005) 15:119–29. doi: 10.3233/VES-2005-15301 16179761

[118] Trinidade A Cabreira V Goebel JA Staab JP Kaski D Stone J. Predictors of persistent postural-perceptual dizziness (PPPD) and similar forms of chronic dizziness precipitated by peripheral vestibular disorders: a systematic review. J Neurol Neurosurg Psychiatry. (2023) 94:904–15. doi: 10.1136/jnnp-2022-330196, PMID: 36941047

[119] Tjernström F Fransson PA Holmberg J Karlberg M Magnusson M. Decreased postural adaptation in patients with phobic postural vertigo--an effect of an "anxious" control of posture? Neurosci Lett. (2009) 454:198–202. doi: 10.1016/j.neulet.2009.03.020, PMID: 19429083

[120] Ahmadi SA Vivar G Frei J Nowoshilow S Bardins S Brandt T. Towards computerized diagnosis of neurological stance disorders: data mining and machine learning of posturography and sway. J Neurol. (2019) 266:108–17. doi: 10.1007/s00415-019-09458-y, PMID: 31286203

[121] Norouzian P Horslen BC Martens KAE. The effects of trait and state anxiety on gait in healthy young adults. Exp Brain Res. (2024) 242:819–28. doi: 10.1007/s00221-024-06800-3, PMID: 38456925

[122] Wuehr M Brandt T Schniepp R. Distracting attention in phobic postural vertigo normalizes leg muscle activity and balance. Neurology. (2017) 88:284–8. doi: 10.1212/WNL.0000000000003516, PMID: 27974646

[123] Best C Tschan R Stieber N Beutel ME Eckhardt-Henn A Dieterich M. STEADFAST: psychotherapeutic intervention improves postural strategy of somatoform vertigo and dizziness. Behav Neurol. (2015) 2015:456850. doi: 10.1155/2015/456850, PMID: 26843786 PMC4710932

[124] Kobel MJ Wagner AR Merfeld DM. Recurrence quantification analysis of postural sway in patients with persistent postural perceptual dizziness. Front Rehabil Sci. (2023) 4:1142018. doi: 10.3389/fresc.2023.1142018, PMID: 37576917 PMC10415033

[125] Yagi C Kimura A Horii A. Persistent postural-perceptual dizziness: a functional neuro-otologic disorder. Auris Nasus Larynx. (2024) 51:588–98. doi: 10.1016/j.anl.2023.12.008, PMID: 38552422

[126] Azami M Fushiki H Tsunoda R Kamo T Ogihara H Tanaka R. Clinical features of persistent postural-perceptual dizziness with isolated otolith dysfunction as revealed by VEMP and vHIT findings. Front Neurol. (2023) 14:1129569. doi: 10.3389/fneur.2023.1129569, PMID: 37006499 PMC10060848

[127] Murofushi T Nishimura K Tsubota M. Isolated otolith dysfunction in persistent postural-perceptual dizziness. Front Neurol. (2022) 13:872892. doi: 10.3389/fneur.2022.872892, PMID: 35481262 PMC9038172

[128] Curthoys IS Burgess AM Manzari L. The evidence for selective loss of otolithic function. Semin Neurol. (2020) 40:33–9. doi: 10.1055/s-0039-3402064, PMID: 31887751

[129] Oane I Barborica A Chetan F Donos C Maliia MD Arbune AA. Cingulate cortex function and multi-modal connectivity mapped using intracranial stimulation. NeuroImage. (2020) 220:117059. doi: 10.1016/j.neuroimage.2020.117059, PMID: 32562780

[130] Aoki M Sakaida Y Tanaka K Mizuta K Ito Y. Evidence for vestibular dysfunction in orthostatic hypotension. Exp Brain Res. (2012) 217:251–9. doi: 10.1007/s00221-011-2989-0, PMID: 22205233

[131] Yagi C Morita Y Yamagishi T Ohshima S Izumi S Takahashi K. Changes in functional connectivity among vestibulo-visuo-somatosensory and spatial cognitive cortical areas in persistent postural-perceptual dizziness: resting-state fMRI studies before and after visual stimulation. Front Neurol. (2023) 14:1215004. doi: 10.3389/fneur.2023.1215004, PMID: 37554393 PMC10406134

[132] Söhsten E Bittar RS Staab JP. Posturographic profile of patients with persistent postural-perceptual dizziness on the sensory organization test. J Vestib Res. (2016) 26:319–26. doi: 10.3233/VES-160583, PMID: 27392836

[133] Ichijo K Oka M Koda K Kamogashira T Kinoshita M Kawahara T. Analysis of postural stability using foam posturography in patients with persistent postural-perceptual dizziness. J Vestib Res. (2024) 34:133–44. doi: 10.3233/VES-230034, PMID: 38073358

[134] Mohebbi A Amiri P Kearney RE. Identification of human balance control responses to visual inputs using virtual reality. J Neurophysiol. (2022) 127:1159–70. doi: 10.1152/jn.00283.2021, PMID: 35353629

[135] Tsuda S Golam M Hou J Nelson R Bernavil P Richardson K. Altered monoaminergic levels, spasticity, and balance disability following repetitive blast-induced traumatic brain injury in rats. Brain Res. (2020) 1747:147060. doi: 10.1016/j.brainres.2020.147060, PMID: 32828734 PMC10424094

[136] Fang Z Huang K Gil C-H Jeong JW Yoo HR Kim HG. Biomarkers of oxidative stress and endogenous antioxidants for patients with chronic subjective dizziness. Sci Rep. (2020) 10:1478. doi: 10.1038/s41598-020-58218-w, PMID: 32001745 PMC6992639

[137] Peng SY Zhuang QX Zhang YX Zhang XY Wang JJ Zhu JN. Excitatory effect of norepinephrine on neurons in the inferior vestibular nucleus and the underlying receptor mechanism. J Neurosci Res. (2016) 94:736–48. doi: 10.1002/jnr.23745, PMID: 27121461

[138] Tang B Jiang W Zhang C Tan H Luo M He Y. Effect of public square dancing combined with serotonin reuptake inhibitors on persistent postural-perceptual dizziness (PPPD) in middle-aged and older women. J Vestib Res. (2024) 34:63–72. doi: 10.3233/VES-230045, PMID: 38043000

