# Supplementary material for: Differential diagnosis of orthostatic dizziness with persistent postural-perceptual dizziness and its underlying mechanisms
Source: Front Neurol. 2025 Oct 1;16:1642869. doi: 10.3389/fneur.2025.1642869 (PMC12520903; doi:10.3389/fneur.2025.1642869)
Supplement: Supplementary file 1 [file Table_1.docx]

Supplementary table 1 Different patterns of OD with diagnosis, treatment, prevention, and prognosis

| **OD patterns** | **epidemiology** | **clinical features** | **Accompani ed diseases** | **Pathophysiology** | **Diagnostic examination** | **Diagnostic criteria** | **treatment** | **prevention** | **prognosis** |
| --- | --- | --- | --- | --- | --- | --- | --- | --- | --- |
| **orthostatic**  **hypotension** | a quarter (24%) of  emergency department  (ED) presentations with  syncope, a fifth (19%) of  older trauma inpatients  and 68% of older general  medicine inpatients | Orthostaticdizzi  ness/vertigo,syn  cope,cognitive  decline,dementia | Review  medications.  optimise causes  autonomic  neuropathy  (HIV/amyloidosis  /diabetes).  Parkinson’s  disease (PD),  dementia with  Lewy  (DLB),  multi-system  atrophy (MSA)  primary  autonomic  failure.diabetic  patients | i) Upon standing, blood pools in  the legs, pelvis and gut, resulting  in reduced venous return to the  heart. ii) Baroreceptors in the  aorta and carotid sinus, and  mechanoceptors in the lungs and  heart detect reduced venous  return to the heart. iii and iv)  Sympathetic nervous system  activation releases noradrenaline  plasma  splanchnic/lower-limb  vasoconstriction. The body’s  ‘orthostasis’. | pressure  standing  from sitting or  head-up tilt test | a sustained reduction  of systolic blood  pressure of at least   1. mmHg or   diastolic blood  pressure of 10  mmHg within 3  minutes of  standing or during  head-up  test;delayed OH,  which is defined as a  sustained  fall of blood pressure  (systolic ≥ 20 mmHg  or diastolic ≥ 10  mmHg) occurring  later than 3 minutes  after standing or  head-up tilt  test;initial OH is  defined as a transient  blood pressure  decrease (systolic >   1. mmHg or   diastolic > 20  mmHg) within 15  seconds of standing,  which may be a | Aim for fluid repletion (2–2.5   1. and 500 mL bolus when   needed. Aim for salt repletion  g/day). Prescribe  compression garments to  include abdominal compression.  Teach  counter-manoeuvres.  Demonstrate head-up sleeping  (by at least 10 degrees).  Volume  fludrocortisone  Sympathomimetics: midodrine  Alternative agents: droxidopa,    atomoxetine and pyridostigmine | Educate on avoiding  triggers  encouraging  self-management,  such as patient diaries.  Patients should be  educated on different  physical  counterpressure  manoeuvres  employ at the onset of  symptoms on standing | It is associated with a  higher risk of  coronary artery  disease, myocardial  infarction, stroke,  falls, fracture, road  accidents and death |

of

bodies

and

into

with

| physiological | | response | to |
| --- | --- | --- | --- |
| standing | is | known | as |

blood

upon

tilt

(4–10

physical

expanders:

and

to

common but

under-recognized

cause of syncope

beta-blockers,Ivabradine,anti-de

pressants and benzodiazepines,

over males by 5:1,

hyperadrenergic,autoimmunity

behavioral therapy, exercise

therapy, group therapy, physical

therapy, occupational

therapy, guided meditation and

yoga; oral fluid intake

a tilt table test. (2)

autonomic

specialists) who care

for these patients.

Symptoms

**postural**

**tachycardia**

**syndrome(POTS)**

**Orthostatic**

**hypoperfusion**

**syndrome**

**orthostatic**

**cerebral**

1% of adolescents;

Females predominate

between 15 and 50 years

About 7.9%

dizziness,

fatigue,

pain,

palpitations,

presyncope,

mental clouding,

nausea,

fatigue,

vision,

dyspnea

orthostatic

dizziness

patients without

orthostatic

anxiety,

blurry

and

and

in

mast

activation

disorder,

Ehlers-Danlos

Syndrome

deconditioning,

and

mutations

norepinephrine

transporters

hypertension and

migraine, CSVD

genetic

cell

of

,

autonomic

probably

neuropathic,

Two

mechanisms

including

vasoconstriction

increase

compliance.

main

of

nervous

CNS

active

peripheral

are

hypovolemic,

and

proposed,

disorder,

cerebral

system;

passive

venous

increase

rate by at least 30

beats per minute

upon assuming an

upright position

Cardiovascular

reflex

included deep

breathing, the

Valsalva

artery using

Transcranial

Doppler.

in

heart

tests

1. A sustained heart

rate elevation of at

least

adults and at least 40

bpm in teens 12–19

years

supine

position

10-min stand test or

Absence

orthostatic

hypotension. (3)

(1)

orthostatic drop

of

flow

(CBFv)

tilt

absence

orthostatic

other

cerebral

test

30

of

to

during

during

age

abnormal

and

bpm

standing

velocity

blood

from

the

(2)

in

of

of

of

or

of

a

,clonidine and

methyldopa,cognitive

Treatment

for

excessive

The clinically

heterogeneous nature

of POTS makes

management

inherently difficult.

aerobic reconditioning

training

it

monitor

addition to heart

and

variables

tilt test.

is

essential

during

CBFv

respiratory

the

to

in

POTS continues to

pose challenges,

from both a

diagnostic and a

therapeutic

standpoint, for

clinicians across

multiple specialties

(cardiology,

neurology,

unknown

orthostatic

intolerance must be

present for at least 6

months

pathophysiological

cerebral vasoconstriction upon

upright posture in the future.

rate, blood pressure,

hypotension.

maneuver, and the

tilt test. CBFv in

the middle cerebral

hypotension,

arrhythmia, vascular

abnormalities,

causes

**Vestibular syncope**

**Benign paroxysmal**

**positional**

**(BPPV)**

**vertigo**

median age was 63 years

(interquartile

range = 54–71 years),

and 33 (62.3%) were

women

a lifetime prevalence of

2.4%,

a

1-year

prevalence of 1.6%, and

1-year incidence of 0.6%.

BPPV accounts for

| 24.1% | of | all | hospital |
| --- | --- | --- | --- |
| visits |  | due | to |

dizziness/vertigo. BPPV

inner

osteoporosis,

ear,

enter

the

semicircular

spontaneously,

canalith

evaluation

as

prevent

syncope.

further

shown

to

be

syncope as a

result of a

vertigo spell

recurrent attacks

of positional

vertigo/dizziness

provoked by

position

changes, and the

characteristic

positional

Meniere’s

Disease,benign

paroxysmal

positional

vertigo,vestibular

paroxysmia,

vestibular

schwannoma,

labyrinthine

concussion, and

inflammatory

multiple cranial

neuropathies;abn

ormal vestibular

function but not

enough to

account for

specific

vestibular

disorders.

head trauma, a

prolonged

recumbent

position, or

various disorders

involving the

a condition in which

vertigo-induced hemodynamic

changes cause syncope,this

change can be arterial

hypertension triggered by a false

downward inertial cue, as

suggested previously, or

hypotension driven by a false

upward inertial cue.

Given the stimulus characteristics

(head motion) and

short-latency action of 100 to 200

msec, this vestibular-driven

reflex regulates autonomic

activity in a

feed-forward manner. In contrast,

the baroreflex system operates

with a feedback mechanism that

controls the autonomic activity.

Calcium carbonate particles in

the utricular otolith membrane of

the elliptical capsule are

dislodged and

canals.When there is a change in

position with respect to gravity,

these particles are moved to

video-oculography,

video head impulse

tests (video-HITs),

cervical and ocular

vestibular evoked

myogenic

potentials

(VEMPs), active

orthostatic blood

pressure, the tilt

table test, and pure

tone audiometry.

Dix-Hallpike and

Roll test maneuver

for diagnosis of

PC-BPPV and

HC-BPPV.

abnormal orthostatic

CBFv.

Please refer to

diagonstic criteria

of syncope.Given its

paroxysmal nature

and the absence of

standard diagnostic

tests, the diagnosis

of vestibular

syncope is primarily

based on an

interview about the

context in which the

syncope occurred.

The gold standard

for diagnosing and

determining the

subtype of BPPV is

the observation of

characteristic

nystagmus triggered

during the positional

Treatment of vestibular

disorders. Please refer to the

management of syncope.

Even though BPPV may resolve

repositioning procedure (CRP)

has been established as the gold

standard for the treatment of

BPPV during the attacks.

Syncope in the setting

of vertigo attacks

requires careful

| managing | underlying |
| --- | --- |
| vestibular | disorders |

can alleviate vertigo

and dizziness and may

A role of preventment

recurrences for

vitamin D in BPPV

has recently been

signifcant. The

vestibular

rehabilitation program

Falls, trauma

BPPV patients show

1-year recurrence

rates of

approximately 20%

and 5-year

recurrence rates of

approximate.BPPV

are at an increased

is most common in

**Chronic anxiety and**

**depressive disorders**

**Bilateral**

**vestibulopathy**

sixties

and

a

disorders;

results

sensation of

dizziness.

motion and

simple

self-report

the

StateTrait

Geriatric

Depression Scale

(GDS), the Beck

Depression

Inventory (BDI),

Anxiety Inventory

(STAI), the

Hospital Anxiety

and Depression

Scale (HADS)

At the bedside,

deficient vestibular

function may be

defined using HITs

and evaluation of

dynamic visual

acuity (DVA) and

balance.

elderly women with a

peak incidence in their

women-to-men ratio of

2.4:1

Patients with dizziness,

using a structured

interview to assess the

prevalence of mental

indicated that 68.2% had

a psychiatric disorder.

Comorbidities among

patients with vestibular

disease and psychiatric

disorder were observed

in 16.0%.

prevalence between 28

and 81 in 100,000 adults.

The prevalence increases

with increasing age (9%

in ≥65 years, 12% in ≥80

years).

nystagmus

elicited by each

positional

maneuver

according to the

subtype and

afected ear.

psychiatric

disorder

The symptoms

include

imbalance,

oscillopsia, and

worsening of

complaints in

darkness

and/or on

uneven ground

hypertension, and

non-apnea sleep

disorders

vestibular

disease(BPPV,M

D,VM,VN)

Meniere’s

disease,

Ototoxicity,

Genetic,

Congenital,

Infectious,

Paraneoplastic,

Migraine,

Gaucher’s

disease,

Nutritional.

different positions in the

semicircular canals leading to the

5-HT decreased

Oscillopsia in BVP is ascribed to

impaired vestibulo-ocular reflex

(VOR) and resultant interruption

of steady fixation of the target on

the fovea during locomotion or

head movements . Patients with

BVP are more prone to

oscillopsia when the corrective

saccades are nonsynchronized.

The unsteadiness and gait

difficulty in BVP particularly get

maneuvers.

The score of

HADS >8

Diagnostic criteria of

bilateral

vestibulopathy

(BVP) by the Barany

Society.Developmen

ts of new diagnostic

tests like HITs and

VEMPs allow more

detailed evaluation

of vestibular

dysfunction in BVP.

SSRIs or SNRIs, or

psychotherapy

Since the vestibular hair cells do

not regenerate in humans,

prevention, correction of the

underlying causes when

available, and vestibular

rehabilitation have been the

mainstay in managing the

patients with BVP.

Vestibular prosthesis, gene

replacement, and stem cell

therapy are emerging

including may reduce

the recurrence rate of

BPPV.

questionnaires can

offer a valid and

efficient means of

detecting a psychiatric

morbidity

Since about 40% of

BVP are associated

with various

neurological

disorders, additional

neurological deficits

should be carefully

sought when

diagnosing BVP.

Falls in people with

Bilateral

risk of depression,

anxiety, falls, and

significant

impairment of

daily activities with

some studies

suggesting an

increased risk of

osteoporosis.

Relatively good

Patients with BVP

have a 9.9- fold

increase of risk for

falls. Vestibular

disorders are

associated with an

increased mortality

of all causes and

deficits in gait and

balance control.

Vestibular prothesis

**Primary orthostatic**

**tremor (OT)**

**Sensory neuropathy**

53.2%-60%

patients

much

likely

more

to

Unique

pathophysiological

orthostatic

tremor,

and

postural

unsteadiness

in

signs

of

and

physical

sharply

peaked

a rare neurological

disease; predominantly

affecting female seniors,

most patients first

develop OT around 60

years of age, the age

range at onset is wide,

from 13 to 85 years.

A family history of OT

was reported in 7%

patients.

Vestibular dysfunction

was encountered in

suffering from peripheral

progressive

neurological

disorder

characterized by

a high frequency

(13-18 Hz)

tremor and

unsteadiness

in the leg

muscles on

standing.

Clinical signs

include the

helicopter sign,

tremor of the

knees, and fine

amplitude

rippling of leg

muscles on

standing

Sensory

neuropathy is

Essential tremor,

parkinsonism,

Non-tumoral

aqueduct

stenosis,

Chronic relapsing

Polyradiculoneur

opathy,Head

trauma,

Graves’ disease,

Small cell lung

cancer, etc.

diabetic

neuropathy,

alcoholic

neuropathy,

worse in darkness

or on uneven ground when the

vision and proprioception cannot

compensate for the deficient

VOR and vestibulospinal reflex.

features of orthostatic tremor

seem to be pontine tegmental

activation, which could relate to

high frequency characteristics of

mesiofrontal deactivation, which

seems to be closely connected to

orthostatic tremor.

The vestibular nerve is a sensory

nerve and is histologically

similar to other peripheral

sensory nerves. It is conceivable

Diagnosis of

orthostatic tremor

was confirmed by

surface

electromyography

(EMG) tremor

recording.

1. type A,

corresponding to

primary OT

without evidence

of dopaminergic

deficit; 2) type B,

corresponding to

primary OT with

dopaminergic

deficits but without

parkinsonism; and

1. type C,

corresponding to

OT associated with

PD.

Vedio-electronysta

gmographic,VEMP

s,vHIT

The diagnosis of OT

is based on history

examination.

However, the

diagnosis should be

confirmed by surface

EMG

recordings revealing

on standing rhythmic

activation of lower

limb muscles at

frequencies between

13 and 18 Hz, and

sometimes higher.

VNG.vHIT,VEMPs,

EMG

therapeutic options for BVP.

clonazepam or gabapentin,

primidone. Alternative drugs

used with varying benefit

include beta-blockers, sodium

valproate, carbamazepine,

baclofen and phenobarbital.

Deep brain stimulation may be

an option in severe, medically

refractory orthostatic tremor.

Treatment for different

peripheral sensory neuropathy

immunomodulatory

vestibulopathy are

common but remain

an understudied

consequence of the

disease.

Physical aids may

offer some sympto

matic relief. For

instance, portable

stools may permit

patients to sit

rather than have to

stand when they are

waiting in line or are

at social events. A

tripod walking stick

could be also helpful

for this purpose.

Furthermore, weight

reduction may be

helpful in overweight

patients.

Given that sensory

neuropathies are, for

the most part, slowly

progressive, those

seems promising in

managing patients

with intractable and

profound BVP.

Progressive and

enigmatic

falls

sensory neuropathy.

**Gait disorders wtih**

**CSVD**

**Neurodegenerative**

**disorders(PD,MSA,PS**

**P)**

worldwide.

Multiple

of

origin.

unknown

White

matter

Refer

to the

PSP

International

of

caloric

responses

balance,

dizziness.

and

dairy

products,

MRI(T1,T2,Flair),

SWI,DWI

MRI, PET, EMG.

a period of

prospective

monitoring

(typically 6–12

months) with a

symptom log

kept by the patient

and serial

examinations

performed by the

clinician before

Reduce of CSVD load

Treatment for PD, MSA and

PSP, etc. Especially focusing on

orthostatic hypotension.

Falls and gradually

decreasing cognitive

PD,MSA and PSP

represent a

fast-growing

neurodegenerative

condition. As early

as after 3 years

from onset, one-third

of patients with

MSA require

walking aids, and

after 5 years, up to

60% of patients

aged 65 or

older,dizziness affected

as many as 17% of

patients with CSVD

Parkinson’s disease is an

age-related disease. PD

has a large effect on

society approximately

6·1 million people who

had been affected

system atrophy (MSA) is

a rare, adult-onset,

progressive

neurodegenerative

disorder with major

manifest with

sensory loss and

pain, or present

as one part of a

multi-factorial

picture, than to

be the sole cause

of dizziness.

Dizziness,unstea

diness

manifest with

dizziness or

unsteadiness

when standing

or walking

before motor

signs can be

detected

on physical

examination.

Between 5 and

30% of MSA

various

demyelinating

diseases,

hereditary

neuropathies

and, frequently, a

polyneuropathy

Risk factors for

cerebrovascular

Parkinson’s

disease(PD),

multiple system

atrophy(MSA),pr

ogressive

supranuclear

palsy(PSP),

cerebellar

degeneration

that pathophysiologic processes

affecting the peripheral sensory

nerves may also affect the

vestibular nerve.

disconnection of cortical

vestibular centers, disconnection

between frontal gait centers and

the basal ganglia, and

disconnection between intended

motor action (efference copy)

and sensory re-afference.

CSVD-mediated dysregulation of

cerebral blood pressure is linked

to dizziness during standing and

walking in elderly patients with

“unexplained” dizziness.

a heterogeneous group of

complex diseases characterized

by neuronal loss and progressive

degeneration of different areas of

the nervous system, a complex

interaction among genetic,

epigenetic, and environmental

factors has been proposed.

hyperintensities,

lacunes, perivascular

spaces, microbleeds,

brain atrophy, small

subcortical infarts

diagnostic criteria

for PD,MSA and

Parkinson and

Movement Disorder

Society

patients with normal

initially may develop

vestibular dysfunction

at a later date.

Screen for CSVD load

and both objective

measures of gait,

dietary factors, such

as the Mediterranean

diet,or consumption of

coffee, tea, alcohol, or

moderate to vigorous

exercise, but not light

exercise, is associated

with a reduced risk of

developing PD. A

natural history study

of MSA showed that

isolated

failure.

autonomic

(93%)

minutes (7%)

or

including

anxiety-related

findings

on

A)

attacks

Recurrent

of

spontaneous

spinning

or

min;

D)

cardiac

diseases

shorter

survival,

for

therapeutic

cardiac

syncope

**Dizziness/vertigo due**

**to cardiac problems**

**Persistent**

**postural-perceptual**

diagnostic challenges.Up

to one-third of MAS

patients presenting with

over the age of 60 (mean

age of 75 years)

prevalence to be 15-20%

among all patients

patients may in

fact present

with urogenital

and

cardiovascular

autonomic

symptoms

several years

before any other

motor symptom

develops.

Recurrent

attacks of

spontaneous

spinning or

non-spinning

vertigo,duration

of CV was only

a few seconds

PPPD manifests

with one or

Bradyarrhythmia,

tachycardia–

bradycardia

syndrome,

complete

atrioventricular

block ischemic

cardiomyopathy

supraventricular

tachycardia,atrial

fibrillation,

angina pectoris,

valvular heart

diseases

BPPV, migraine,

vestibular

heart fails to generate adequate

cardiac output, the brain is

inadequately perfused and

temporarily malfunctions,

leading to a syncopal event.

Transient or small reduction of

brain perfusion caused by

cardiovascular diseases would

develop isolated vertigo without

loss of consciousness.

personality traits as a possible

making a definitive

diagnosis.

vestibular and

cardiac function

tests

There are no

non-spinning vertigo

1. Onset over the

age of 60;

1. Duration of

vertigo less than 1

Documentation of

during an attack of

vertigo; E) Response

to proper treatments

for cardiac diseases;

1. Not better

accounted for by

another diagnosis

Please refer to

diagnostic criteria

Treatment for different cardiac

problems

a highly anxious response to

precipitating events may be the

low vitamin B12

levels (< 367 ng/L)

were associated with

increased frequency

of falls within three

years of onset, and a

lower body mass

index, suggesting a

potential new target

intervention

early diagnosis of

cardiogenic vertigo

anxiety-related

personality traits or a

become wheel

chair-bound.

1-year mortality of

estimates to about

30%

chronic functional

vestibular disorder

pivotal initial pathophysiologic

anxiety and vigilance about acute

disorders may be risk

factors for developing

responses. Alterations in postural

the second most common

among all adults.

months or more.

orientation

assessment

sustaining mechanisms.

It is caused by three types of

closure of types 1 and 2 leaks

tears (type 1), leaking root nerve

**dizziness (PPPD)**

**Intracranial**

**hypotension**

presenting for evaluation

of vestibular symptoms,

making

common

among young adults and

annual incidence of 5%

them

the

diagnoses

most

more symptoms

of

unsteadiness,

non-spinning

vertigo that are

present on most

days

orthostatic

headache

syndrome, some

patients with

dizziness, a

range of further

presentations,

ranging all the

way to coma

or apparent

frontotemporal

dementia

dizziness,

for

three

or

neuritis,

Meniere’s

disease

Meniere’s

disease,

Ehlers-Danlos

syndrome, and

Marfan

syndrome,

CSF-venous

fstulas,Spinal

meningeal

diverticulum.

risk factor and

symptoms

events

control

multi-sensory

reduced

spatial

spinal CSF leaks: Ventral dural

sleeves associated with

as

cortical

strategies,

during

networks

initial

integration,

high

integration

precipitating

and

pathologic

shifts

levels

may

threat

and

be

of

in

of

physical

examination,

laboratory testing,

or

imaging

pathognomonic

PPPD.

Diagnostic criteria

include

pressure<60

H2O

evidence of a CSF

leak on imaging.

diagnostic

that

a

and/or

CSF

mm

are

of

for

postural-perceptual

dizziness (PPPD) in

vestibular

of

Society

lumbar puncture and

spine

MRI

the

MRI,

persistent

Disorders

Barany

head

process in the development of

PPPD,

symptom-specific

might counter this effect.

Minimal

and

the

CSF-venous fstulas are elegant

azygos

transvenous

and

invasive

vein

that

interventions

embolization

system

surgical

early

for

personal

history

PPPD

relevant precipitants.

Risk is higher when

treatment

for more than

10 weeks and when

patients

Around 1/5 of patients

treated

or

surgery

of

with

are

are

or

is

following

anxiety

delayed

develop

usually

family

obese.

either

RIH).

with

for

Complications, such

as subdural

hematoma, cerebral

venous thrombosis,

can lead to a change

in

pattern

potentially

consequences.

the

headache

and

with liquid embolic agents via

meningeal diverticulae (type 2),

and CSF-venous fstulas (type 3).

therapeutic options.

epidural blood patches

life-threatening

symptoms of rebound

hypertension (rebound

intracranial

hypertension

Patients

managed

acetazolamide

some weeks.
